# Phenylacetylglutamine, a Novel Biomarker in Acute Ischemic Stroke

**DOI:** 10.3389/fcvm.2021.798765

**Published:** 2021-12-23

**Authors:** Fang Yu, Xi Li, Xianjing Feng, Minping Wei, Yunfang Luo, Tingting Zhao, Bo Xiao, Jian Xia

**Affiliations:** ^1^Department of Neurology, Xiangya Hospital, Central South University, Changsha, China; ^2^Clinical Research Center for Cerebrovascular Disease of Hunan Province, Central South University, Changsha, China; ^3^National Clinical Research Center for Geriatric Disorders, Xiangya Hospital, Central South University, Changsha, China

**Keywords:** ischemic stroke, metabolomics, biomarkers, phenylacetylglutamine, microbiota

## Abstract

**Background:** To discover novel metabolic biomarkers of ischemic stroke (IS), we carried out a two-stage metabolomic profiling of IS patients and healthy controls using untargeted and targeted metabolomic approaches.

**Methods:** We applied untargeted liquid chromatography-mass spectrometry (LC-MS) to detect the plasma metabolomic profiles of 150 acute IS patients and 50 healthy controls. The candidate differential microbiota-derived metabolite phenylacetylglutamine (PAGln) was validated in 751 patients with IS and 200 healthy controls. We evaluated the associations between PAGln levels and the severity and functional outcomes of patients with IS. Clinical mild stroke was defined as the National Institutes of Health Stroke Scale (NIHSS) score 0–5, and moderate-severe stroke as NIHSS score >5. A favorable outcome at 3 months after IS was defined as the modified Rankin Scale (mRS) score 0–2, and unfavorable outcome as mRS score 3–6.

**Results:** In untargeted metabolomic analysis, we detected 120 differential metabolites between patients with IS and healthy controls. Significantly altered metabolic pathways were purine metabolism, TCA cycle, steroid hormone biosynthesis, and pantothenate and CoA biosynthesis. Elevated plasma PAGln levels in IS patients, compared with healthy controls, were observed in untargeted LC-MS analysis and confirmed by targeted quantification (median 2.0 vs. 1.0 μmol/L; *p* < 0.001). Patients with moderate-severe stroke symptoms and unfavorable short-term outcomes also had higher levels of PAGln both in discovery and validation stage. After adjusting for potential confounders, high PAGln levels were independently associated with IS (OR = 3.183, 95% CI 1.671–6.066 for the middle tertile and OR = 9.362, 95% CI 3.797–23.083 for the highest tertile, compared with the lowest tertile) and the risk of unfavorable short-term outcomes (OR = 2.286, 95% CI 1.188–4.401 for the highest tertile).

**Conclusions:** IS patients had higher plasma levels of PAGln than healthy controls. PAGln might be a potential biomarker for IS and unfavorable functional outcomes in patients with IS.

## Introduction

Stroke is a great threat to public health globally, especially in developing countries. The China Stroke Statistics published in 2020 showed that stroke ranked third among the leading causes of death in China, and 81.9% of stroke inpatients had ischemic strokes (IS) (1). Despite decades of substantial basic and clinical studies, the etiology of IS has not been fully elucidated, and only a few effective treatments are available. Therefore, it is essential to discover new circulating biomarkers to help understand the pathogenesis of IS.

Unlike well-studied genomics and proteomics, metabolomics technologies have only made a major shift in the last two decades. Metabolomics profiling can provide high-throughput data and can identify small metabolites (<1,500 Da) that may offer novel insight into biological events (2). Metabolomics has emerged as a promising approach for identifying potential biomarkers of cerebrovascular disease. Metabolites derived from gut-microbiota, such as trimethylamine N-oxide (TMAO), which is the most studied gut microbiota-dependent metabolite, has been reported to be associated with IS and atherosclerosis (3–6). Unfortunately, most studies have limited sample sizes and lack independent validation.

In this study, we initially conducted an untargeted metabolomic analysis to uncover plasma metabolites that were most strongly distinguished between IS patients and controls. We found that levels of PAGln, another microbiota-derived metabolite associated with cardiovascular disease (CVD) (7), were elevated in patients with IS. These results were confirmed in a validation sample set using absolute quantitation. Additionally, we investigated the associations between PAGln levels and the severity and functional outcomes of patients with IS.

## Materials and Methods

### Study Participants

**Figure 1:**
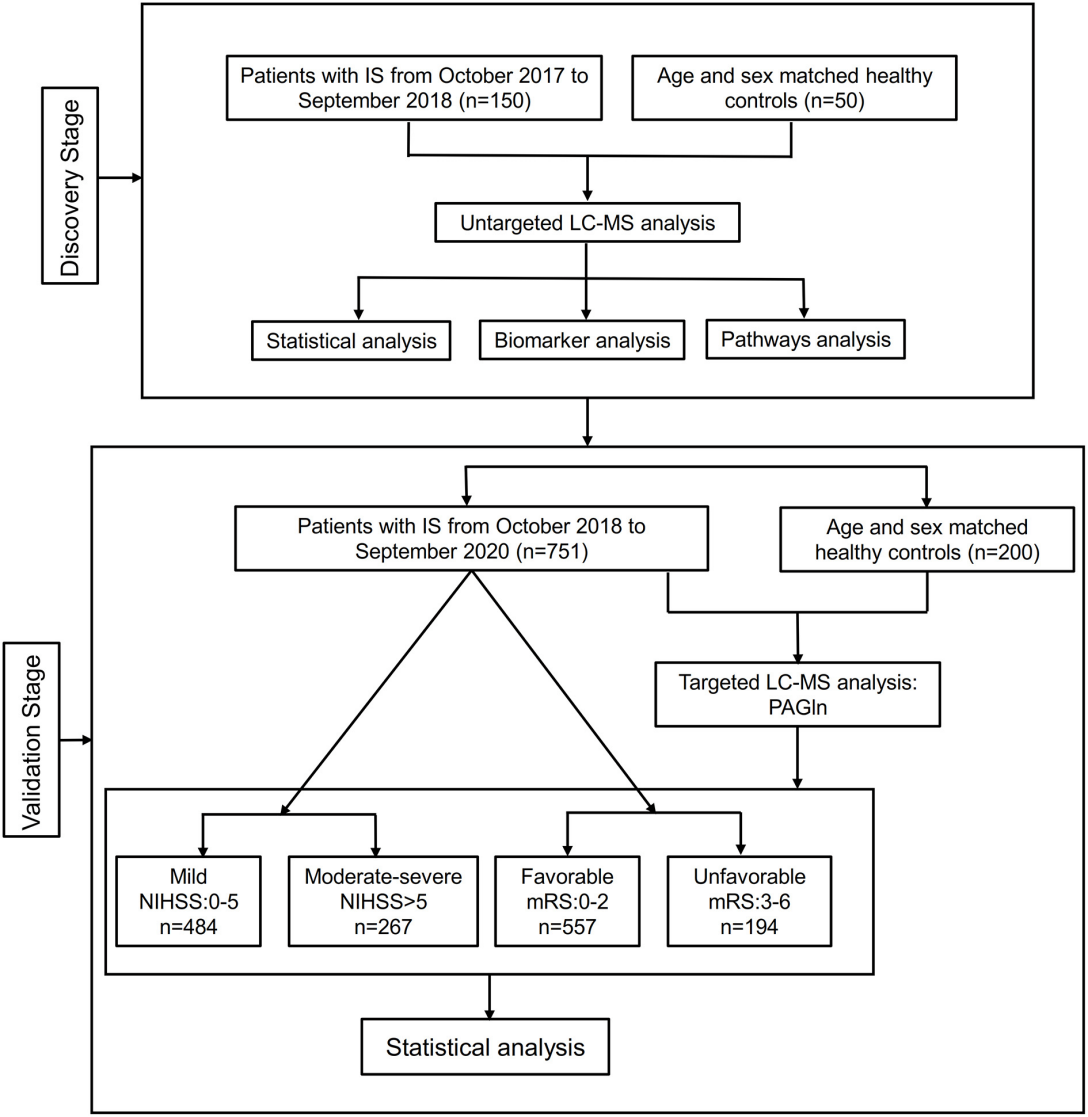
The flowchart of the study design. IS, ischemic stroke; LC-MS, liquid chromatography-mass spectrometry; PAGln, phenylacetylglutamine; NIHSS, NIH Stroke Scale; mRS, modified Rankin Scale.

From October 2017 to September 2020, 150 IS patients and 50 healthy controls for untargeted metabolomic discovery and 751 IS patients and 200 controls for targeted validation were recruited from Xiangya Hospital, Central South University. The flowchart of this study is shown in Figure 1. The specific inclusion criteria of patients with IS included (1) admission within 7 days of onset, (2) age ≥18 years, (3) confirmed infarction assessed diffusion-weighted imaging (DWI), and (4) complete clinical examination and imaging data. The exclusion criteria were as follows: (1) pre-onset modified Rankin Scale (mRS) score >2, (2) impaired hepatic or renal function, (3) infectious diseases (pulmonary infection, urinary system infection, gastrointestinal tract infection, etc.) and the usage of antibiotics, probiotics within 30 days before admission; (4) other autoimmune diseases, and (5) malignancy. Healthy controls were selected from the Health Management Center of Xiangya Hospital during the same period. These participants were in good general health. The inclusion criteria of controls were the absence of stroke. The exclusion criteria for control group were identical to the exclusion criteria in IS patients. The study protocol was approved by the National Clinical Research Center for Geriatric Disorders of Xiangya Hospital and the Ethics Committee of Xiangya Hospital. All participants or their families provided written, signed informed consent.

### Neuroimaging

All patients completed brain 1.5T or 3.0T MRI or CT scans, as well as angiographic examinations. Healthy controls were also assessed using cranial MRI and angiography to ensure that they did not have infarcts or artery stenosis. Two neurologists (Xi Li and Xianjing Feng) independently evaluated the images.

### Clinical Examination

At the time of enrollment, we interviewed all participants face-to-face to collect information on age, sex, admission blood pressure and vascular risk factors. Vascular risk factors such as hypertension, diabetes mellitus, hyperlipidemia, coronary heart disease (CAD), smoking and drinking status were defined as described previously (8). Clinical biochemical data were obtained from the Department of Clinical Laboratory of our hospital. The estimated glomerular filtration rate (eGFR) was calculated by the Chronic Kidney Disease Epidemiology Collaboration (CKD-EPI) equation (9).

### Stroke Severity and Short-Term Outcomes Measures

The neurological deficits, including consciousness, facial and limb paralysis, language, and other aspects of IS patients are assessed by the National Institutes of Health Stroke Scale (NIHSS) score (15 items, range 0–42 points) (10). The mRS score (range 0–6 points) which shows the disability in daily life (11) is often used for the evaluation of primary outcomes after stroke onset. The NIHSS score on admission and the mRS score at 3 months after stroke onset are used to measure the severity and short-term outcomes of acute IS, respectively. Upon admission, the NIHSS scores were evaluated by certified neurologists at the clinical examination. We defined mild stroke as an NIHSS score 0–5, and moderate to severe stroke as an NIHSS score >5 (12). We evaluated the mRS scores of patients at 3 months after stroke onset via telephone follow-up or outpatient visits. The mRS score of 0–2 indicated favorable functional outcomes, while 3–6 indicated unfavorable outcomes (11).

### Sample Collection and Preparation

Blood samples (3 mL) were drawn after overnight fasting. The collected samples were centrifuged at 3,000 rpm for 15 min at 4°C. We stored these plasma samples in refrigerators at −80°C. Multiple freeze-thaw cycles were avoided in all samples until analysis.

### Untargeted Metabolomic Profiling of Plasma Using High-Resolution Liquid Chromatography-Mass Spectrometry (LC-MS)

First, a 100 μL plasma sample was deproteinized with 400 μL acetonitrile and methanol mixture (1:1, v/v), vortexed for 30 s, sonicated for 10 min, and centrifuged at 12,000 rpm for 15 min at 4°C; then, the supernatants of the plasma sample were collected and transferred to a new 1.5 mL centrifuge tube and dried with nitrogen. The sample was then dissolved in 100 μL of acetonitrile and water (1:1, v/v), vortexed for 30 s, sonicated for 5 min, and centrifuged at 12,000 rpm for 15 min at 4°C. Finally, the supernatant was transferred to an autosampler vial. LC-MS analysis was performed using an AB SCIEX TripleTOF 5600 system (AB SCIEX, Foster City, CA, USA). Separations were conducted on a Waters ACQUITY UPLC T3 column (2.1 × 100 mm; 1.8 μm). Mobile phase A consisted of 0.1% aqueous formic acid, and phase B consisted of acetonitrile containing 0.1% formic acid. The injection volume was 2 μL for the positive electrospray ionization (ESI) mode and negative ESI mode. The elution program was as follows: 0–2 min, 5% B; 2–5 min, 5–70% B; 5–14 min, 70–90% B; 14–16 min, 90–100 % B; 16–22 min, 100 % B; 22–22.1 min, 100–5% B; 22.1–25 min, 5% B. The flow rate was 0.3 mL/min. MS and MS/MS data were analyzed in the information-dependent acquisition (IDA) mode. The mass ranged from 50 to 1,000 m/z. The curtain gas (CUR) = 35 psi, ion spray voltage floating (ISVF) = 5,500/−4,500 (+/–) V, declustering potential (DP) = 80 V, collision energy (CE) = 40 ± 20 V, ion source gas1 (GS1) = 55 psi, ion source gas2 (GS2) = 55 psi, and temperature (TEM) = 550°C. To fulfill the criteria of IDA and avoid the omission of small metabolites, dynamic background subtraction (DBS) was chosen to screen the profile.

### Targeted LC-MS Analysis of PAGln

Plasma levels of PAGln were quantified in positive mode with multiple reaction monitoring (MRM) mode, as previously described (7). Briefly, 2 μL of 1 ppm D5-PAGln (CDN Isotopes, Cat # D-6900) and 150 μL of ice-cold methanol were added to 48 μL of diluted plasma (5 μL plasma and 43 μL ddH2O), vortexed for 1 min, and then centrifuged at 21,000 × g at 4°C for 15 min. The supernatant was transferred to a clean vial for testing. LC-MS analysis was performed using the AB SCIEX Qtrap 6500 system (AB SCIEX, Foster City, CA, USA). The separation was conducted on an Acquity UPLC BEH C18 column (50 × 2.1 mm, 1.7 μm). Mobile phase A was 0.1% acetic acid in water and mobile phase B was 0.1% acetic acid in acetonitrile. The elution program was as follows: 0–2.5 min, 5% B; 2.5–3 min, 5–95% B; 3–3.1 min, 95–5% B; 3.1–4 min, 5% B. The injection volume was 1 μL and the flow rate was 0.3 mL/min. The ion source parameters were as follows: CUR, 40 psi; ISVF, 5,500 V; GS1, 60 psi; GS2, 55 psi; and TEM, 550°C. The quality control (QC) samples with low, medium, and high concentrations (5, 50, 400 ng/ml) of PAGln (Santa Cruz Biotechnology, Cat # SC-212551A) were measured every twenty samples. The intra-day coefficients of variation ranged from 0.8 to 1.4% and the inter-day coefficients of variation ranged from 4.0 to 6.0%. The extracted ion chromatograms of PAGln and the isotope standard (D5-PAGln) were shown in Supplementary Figure S5A. The calibration curve (Supplementary Figure S5B) exhibited good linearity (r^2^ = 0.99913) with concentrations ranging from 1 to 500 ng/ml.

### Data Processing and Metabolomics Data Analysis

Untargeted LC-MS raw data were converted into mzXML files using ProteoWizard and then imported into XCMSplus software for mzMatch, peak alignment, calibration, and peak retention time (13). Differential metabolites were identified by database matching using the HR MS/MS library (AB Sciex, Forster City, CA, USA) and MetDNA (http://metdna.zhulab.cn/) (14). We used MetaboAnalyst 5.0 (https://www.metaboanalyst.ca) for metabolite data analysis. First, the metabolite intensity values were log-transformed and autoscaled (mean-centered and divided by the standard deviation of each variable). Partial least squares discriminant analysis (PLS-DA) was performed to inspect group disparity. Variable influence on projection (VIP) values for each variable were obtained from the PLS-DA model. The differences of normalized peak intensities of metabolites between two groups were analyzed by Student's *t* test, followed by false discovery rate (FDR) adjustment for multiple comparisons. Metabolites with VIP >1, *p* < 0.05 after FDR adjustment were regarded as differential biomarkers. Metabolic pathway analysis was carried out using the Pathway Analysis module of MetaboAnalyst 5.0, and the pathway library of Kyoto Encyclopedia of Genes and Genomes (KEGG) (https://www.kegg.jp). The multivariable ROC (Receiver operating characteristic curve) analyses were used to identify the abilities of the candidate metabolites in discriminating IS from controls and the area under the ROC curves (AUC) was calculated (Biomarker Analysis module of the MetaboAnalyst 5.0). Monte-Carlo cross validation was used for the generation of multivariable ROC curves. Important metabolites (top 5, 10, 15, 25, 50, 100) were evaluated by 2/3 of all samples and validated in the remaining 1/3 samples. Performance was calculated after the procedures were repeated many times. The SCIEX OS-Q 2.0 (https://sciex.com/products/software/analyst-software) was used for targeted metabolomics data analysis.

### Statistical Analysis

We used SPSS version 22.0 (IBM SPSS, Chicago, IL, USA) for clinical data analysis. We first tested whether the data were normally distributed using the Kolmogorov-Smirnov test. Non-normal distributed continuous data were reported as median (interquartile range) in this study and were assessed using the Mann-Whitney *U* test. Categorical data were presented as numbers (n) and percentages (%) and were tested using the χ2 test. Binary logistic regression analyses were performed to identify independent factors associated with the onset, severity and outcomes of IS. To better interpret the results, PAGln levels were divided into tertiles and logistic analyses were performed in three models (Model 1: adjusted for age and sex; Model 2: adjusted for age, sex, hypertension, diabetes mellitus, hyperlipidemia, CAD, smoking and drinking status; Model 3: adjusted for the variables in Model 2 and other variables that were statistically significant in **Table 2**). Results of logistic regression analyses were expressed as adjusted odds ratios (ORs) and 95% confidence intervals (CIs). We used Spearman correlation analysis to evaluate the relationship between the top 20 metabolites detected in untargeted metabolomics and PAGln levels quantified in targeted metabolomics and other clinical parameters. ROC analyses were generated using MedCalc 19.4.1 (MedCalc Inc., Mariakerke, Belgium) to evaluate the abilities of PAGln in discriminating IS from controls, moderate-severe stroke from mild stroke and unfavorable stroke outcome from favorable outcome. A two-tailed *p* < 0.05 was considered statistically significant. Correlation heatmaps were performed on an R-based online platform (https://www.omicstudio.cn/tool/) and other data were plotted using GraphPad Prism version 8.0.

## Results

### Characteristics of all Participants

In this study, 150 patients with IS and 50 healthy controls were included in the discovery stage. In the validation stage, there were 751 patients with IS and 200 healthy controls. The clinical and demographic information of these participants are presented in Tables 1, 2. Patients with IS and healthy controls were frequency matched for age and sex.

**Table 1:** General characteristics of ischemic stroke patients and healthy controls in discovery stage.

	**IS (*n =* 150)**	**Control (*n =* 50)**	***p***
Age (years)	60 (53–68)	57 (55–66)	0.696
Sex (male, N, %)	100 (66.7%)	31 (62.0%)	0.548
HBP (N, %)	100 (66.7%)	20 (40.0%)	<0.001
DM (N, %)	47 (31.3%)	4 (8.0%)	0.001
Hyperlipidemia (N, %)	24 (16.0%)	24 (48.0%)	<0.001
CAD (N, %)	28 (18.7%)	9 (18.0%)	0.916
Smoking (N, %)	74 (49.3%)	14 (28.0%)	0.008
Drinking (N, %)	59 (39.3%)	10 (20.0%)	0.013
Admission NIHSS	4 (2–7)	NA	NA
mRS at 3 months	2 (1–3)	NA	NA
SBP (mmHg)	142 (127–154)	130 (120–139)	<0.001
DBP (mmHg)	83 (74–91)	80 (72–85)	0.074
WBC (×10^9^/L)	6.6 (5.4–8.2)	5.8 (4.9–6.9)	0.016
Platelet (×10^9^/L)	192.0 (148.0–225.8)	205.0 (164.2–233.5)	0.126
BUN (mmol/L)	5.0 (4.2–6.0)	4.6 (4.0–6.2)	0.784
UA (μmol/L)	337.0 (279.0–394.6)	354.0 (289.4–434.4)	0.191
Creatinine (μmol/L)	79.9 (68.0–89.0)	74.2 (67.2–83.8)	0.172
eGFR (ml/min per 1.73 m^2^)	84.9 (70.2–95.6)	91.0 (76.8–98.7)	0.231
TG (mmol/L)	1.6 (1.1–2.0)	1.6 (1.3–2.4)	0.374
TC (mmol/L)	4.1 (3.4–4.8)	4.9 (4.2–5.5)	<0.001
HDLC (mmol/L)	1.02 (0.83–1.18)	1.23 (0.99–1.40)	<0.001
LDLC (mmol/L)	2.4 (1.9–2.9)	3.1 (2.6–3.6)	<0.001
FBG (mmol/L)	6.0 (5.1–7.8)	5.1 (4.7–5.8)	0.01
HbA1c (%)	6.0 (5.6–7.2)	5.8 (5.5–6.0)	0.114
Homocysteine (μmol/L)	13.4 (11.2–17.4)	12.8 (10.5–13.6)	0.042

*IS, ischemic stroke; HBP, hypertension; DM, Diabetes mellitus; CAD, coronary artery disease; NIHSS, National Institutes of Health Stroke Scale; mRS, modified Rankin Scale; SBP, systolic blood pressure, DBP, diastolic blood pressure; WBC, white blood cell; BUN, blood urea nitrogen; UA, uric acid; eGFR, estimated glomerular filtration rate; TG, triglyceride; TC, total cholesterol; HDLC, high density lipoprotein cholesterol; LDLC, low density lipoprotein cholesterol; FBG, fasting blood glucose; HbA1c, glycosylated hemoglobin A1c*.

**Table 2:** Baseline characteristics of ischemic stroke patients and healthy controls in validation stage.

	**Disease status**			**Stroke severity**			**Short–term outcome**		
**Variables**	**Control** **(*n =* 200)**	**IS** **(*n =* 751)**	***p***	**Mild** **(*n =* 484)**	**Moderate to severe** **(*n =* 267)**	***p***	**Favorable** **(*n =* 557)**	**Unfavorable** **(*n =* 194)**	***p***
Age (years)	61 (52–67)	61 (52–69)	0.620	60 (51–68)	61 (53–70)	0.088	59 (51–67)	65 (56–72)	<0.001
Sex (male, N, %)	129 (64.8%)	516 (68.7%)	0.297	342 (70.7%)	174 (65.2%)	0.120	398 (71.5%)	118 (60.8%)	0.006
HBP (N, %)	83 (41.7%)	530 (70.6%)	<0.001	326 (67.4%)	204 (76.4%)	0.009	380 (68.2%)	150 (77.3%)	0.017
DM (N, %)	32 (16.1%)	217 (28.9%)	<0.001	129 (26.7%)	88 (33.0%)	0.068	142 (25.5%)	75 (38.7%)	<0.001
Hyperlipidemia (N, %)	79 (39.5%)	200 (26.6%)	<0.001	131 (27.1%)	69 (25.8%)	0.717	147 (26.4%)	53 (27.3%)	0.801
CAD (N, %)	16 (8.0%)	137 (18.2%)	<0.001	87 (18.0%)	50 (18.7%)	0.799	86 (15.4%)	51 (26.3%)	<0.001
Smoking (N, %)	79 (39.7%)	363 (48.3%)	0.030	245 (50.6%)	118 (44.2%)	0.326	283 (50.8%)	80 (41.2%)	0.022
Drinking (N, %)	71 (35.5%)	274 (36.5%)	0.797	176 (36.4%)	98 (36.7%)	0.092	211 (37.9%)	63 (32.5%)	0.178
Admission NIHSS	NA	4 (1–7)	NA	2 (1–4)	9 (7–11)	<0.001	3 (1–5)	8 (5–11)	<0.001
mRS at 3 months	NA	2 (1–3)	NA	1 (1–2)	3 (2–3)	<0.001	1 (1–2)	3 (3–4)	<0.001
SBP (mmHg)	132 (120–148)	142 (129–156)	<0.001	141 (128–154)	143 (130–159)	0.022	142 (128–155)	143 (129–159)	0.241
DBP (mmHg)	83 (78–90)	82 (74–92)	0.237	82.0 (73.0–92.0)	83.0 (75.0–92.0)	0.231	82 (74–92)	82 (74–91)	0.855
PAGln (μmol/L)	1.0 (0.5–1.9)	2.0 (1.2–3.3)	<0.001	1.9 (1.0–3.2)	2.3 (1.3–3.5)	0.007	1.9 (1.1–2.9)	2.5 (1.6–4.0)	<0.001
WBC (×10^9^/L)	6.0 (5.1–7.4)	6.7 (5.6–8.1)	0.001	6.5 (5.4–7.7)	7.2 (5.8–8.9)	<0.001	6.6 (5.5–7.9)	7.2 (5.7–8.9)	0.002
Platelet (×10^9^/L)	213.0 (188.2–246.0)	203.0 (164.0–242.8)	0.026	202.0 (164.0–239.0)	205.0 (167.8–254.8)	0.249	204.0 (164.8–241.2)	199.0 (162.0–247.5)	0.536
BUN (mmol/L)	4.8 (3.9–5.8)	5.0 (4.1–6.1)	0.015	5.0 (4.0–6.1)	5.1 (4.2–6.4)	0.209	4.9 (4.0–6.0)	5.2 (4.2–6.6)	0.015
UA (μmol/L)	334.3 (282.6–392.9)	321.5 (268.2–386.9)	0.048	329.5 (276.6–393.5)	305.0 (238.7–377.0)	<0.001	326.9 (272.8–389.8)	307.0 (250.5–381.8)	0.016
Creatinine (μmol/L)	82.0 (69.8–95.6)	83.0 (71.8–94.0)	0.650	83.9 (73.0–95.0)	81.0 (69.9–90.8)	0.016	83.0 (72.0–95.0)	82.0 (70.4–92.9)	0.393
eGFR (ml/min per1.73 m^2^)	83.9 (69.8–95.1)	84.2 (70.0–94.7)	0.745	83.2 (69.8–94.2)	85.6 (71.1–95.6)	0.251	85.3 (70.8–95.9)	80.9 (65.9–92.1)	0.004
TG (mmol/L)	1.6 (1.2–2.3)	1.5 (1.1–2.1)	0.029	1.5 (1.1–2.0)	1.4 (1.1–2.2)	0.516	1.5 (1.1–2.1)	1.5 (1.1–2.2)	0.652
TC (mmol/L)	5.1 (4.3–5.9)	4.1 (3.4–5.0)	<0.001	4.0 (3.4–4.9)	4.3 (3.4–5.2)	0.047	4.1 (3.4–5.0)	4.2 (3.4–4.9)	0.686
HDLC (mmol/L)	1.21 (1.05–1.41)	0.99 (0.84–1.17)	<0.001	0.97 (0.84–1.15)	1.02 (0.87–1.21)	0.028	0.97 (0.84–1.16)	1.02 (0.87–1.21)	0.133
LDLC (mmol/L)	3.2 (2.6–3.7)	2.5 (2.0–3.2)	<0.001	2.5 (2.0–3.1)	2.6 (2.0–3.4)	0.084	2.5 (2.0–3.2)	2.6 (2.0–3.1)	0.770
FBG (mmol/L)	5.5 (5.1–6.1)	5.6 (4.9–7.1)	0.162	5.4 (4.8–6.6)	6.0 (5.1–8.1)	<0.001	5.5 (4.9–6.8)	5.9 (5.1–7.9)	0.002
HbA1c (%)	5.9 (5.5–6.5)	5.9 (5.5–6.8)	0.431	5.8 (5.5–6.4)	5.9 (5.5–7.4)	0.037	5.8 (5.5–6.6)	6.1 (5.6–7.3)	0.007
Homocysteine (μmol/L)	13.0 (10.9–15.2)	13.3 (11.2–16.6)	0.059	13.8 (11.4–17.1)	12.7 (10.8–15.5)	0.005	13.5 (11.3–16.7)	12.9 (10.8–16.4)	0.157

*IS, ischemic stroke; HBP, hypertension; DM, Diabetes mellitus; CAD, coronary artery disease; NIHSS, NIH Stroke Scale; SBP, systolic blood pressure, DBP, diastolic blood pressure; PAGln, phenylacetylglutamine; WBC, white blood cell; BUN, blood urea nitrogen; UA, uric acid; eGFR, estimated glomerular filtration rate; TC, total cholesterol; TG, triglyceride; HDLC, high density lipoprotein cholesterol; LDLC, low density lipoprotein cholesterol; FBG, fasting blood glucose; HbA1c, glycosylated hemoglobin A1c*.

### Untargeted Metabolomics Profiling Detected PAGln Accumulation in IS Patients

Representative LC-MS total ion current (TIC) chromatograms of the QC plasma samples were shown in Supplementary Figure S1. A total of 571 metabolites were detected in all plasma samples and used in the subsequent multivariable analysis.

**Figure 2:**
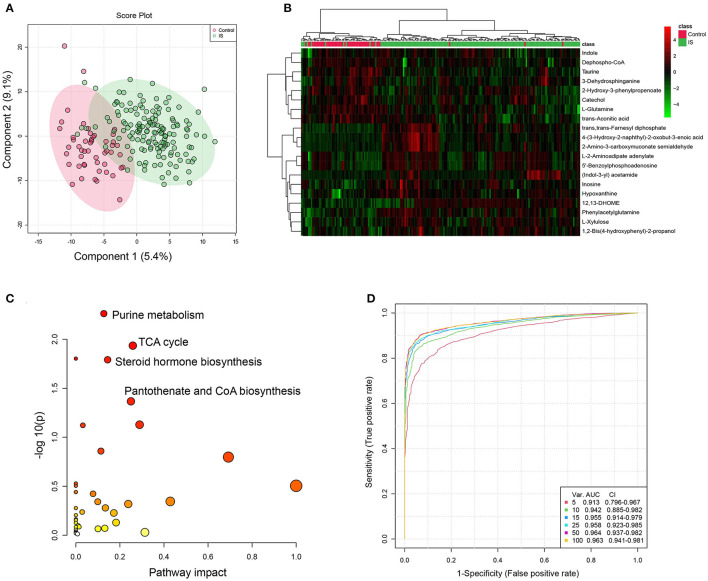
Multivariable statistical analysis and pathway analysis between IS patients and controls. **(A)** PLS-DA plots shows clear separation of healthy controls (red) and ischemic stroke (IS) patients (green). **(B)** Heatmap of top 20 differential metabolites (ranked by VIP values) between IS patients and healthy controls. **(C)** Metabolic pathways for IS patients relative to healthy controls. The X-axis represents pathway impact, and the Y-axis represents -log10 (p). **(D)** The multivariable ROC curves for sets of metabolites. Monte-Carlo cross validation was used for the generation of multivariable ROC curves. Important metabolites (top 5, 10, 15, 25, 50, 100) were evaluated by 2/3 of all samples and validated in the remaining 1/3 samples. The metabolites numbers and AUC (95% CI) of six models are presented. Classification method (Random Forests) and feature ranking method (Univariable AUC) were used. IS, ischemic stroke; PLS-DA, partial least squares discriminant analysis; VIP, Variable influence on projection; ROC, receiver operating characteristic curve; AUC, area under the curve.

**Figure 3:**
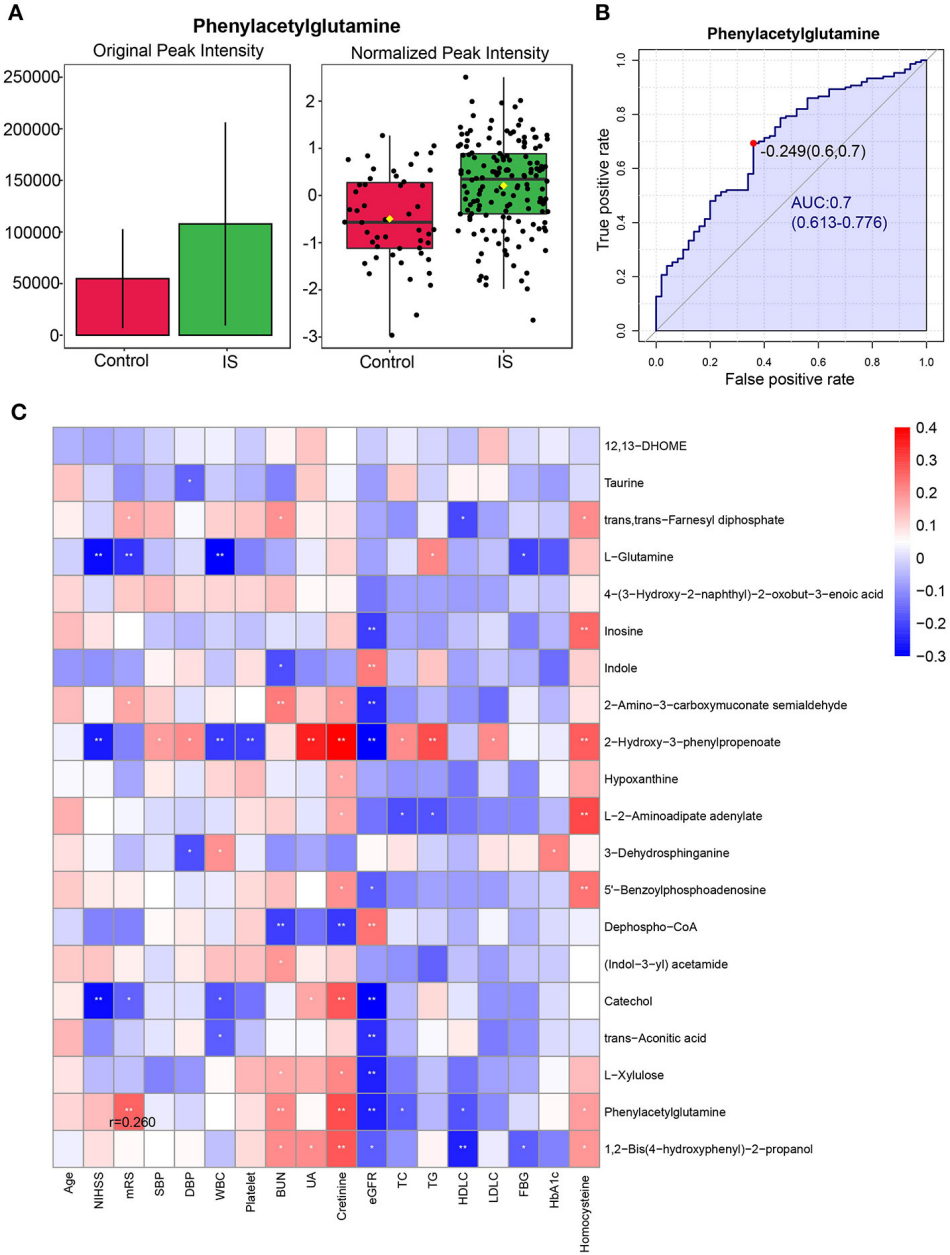
Phenylacetylglutamine (PAGln) levels in discovery stage. **(A)** The original peak intensity and normalized peak intensity of plasma phenylacetylglutamine in IS patients (*n* = 150) and the healthy controls (*n* = 50) (*p* <0.001) (Student's T test). **(B)** The ROC analysis of plasma relative phenylacetylglutamine levels to differentiated patients with IS from healthy controls. **(C)** Correlation heatmap of the top 20 metabolites ranked by VIP values and clinical parameters. IS, ischemic stroke; ROC, receiver operating characteristic curve; VIP, Variable influence on projection.

The PLS-DA model suggested that metabolome profiles differed between patients with IS and healthy controls (Figure 2A). Based on VIP and P values after FDR adjustment (VIP >1, *p*_FDR_ < 0.05), we detected 120 differential metabolites (shown in Supplementary Table S1). Figure 2B shows the heatmap of the top 20 differential metabolites based on VIP values. Pathway analysis showed that purine metabolism, TCA cycle, steroid hormone biosynthesis, and pantothenate and CoA biosynthesis were significantly altered (Figure 2C and Supplementary Table S2). The multivariable ROC analyses were showed in Figure 2D, and the legend included feature numbers, AUCs and the 95% CIs of the six models. The maximum value of AUC was 0.963 (95% CI: 0.941–0.981) when top 100 metabolites were selected. The most important metabolites of selected Model 1 (5 features), Model 2 (10 features), Model 3 (15 features), and Model 4 (25 features) of the multivariable ROC curves were shown in Supplementary Figure S3. Among differential metabolites, the gut microbiota-derived metabolite, phenylacetylglutamine (PAGln) (HMDB0006344, Supplementary Figure S4) in the positive ion mode, exhibited an increase in IS patients (*p* < 0.001, VIP = 1.923, fold change = 1.968, AUC = 0.700), suggesting its potential as a biomarker for IS patients (Supplementary Table S1 and Figures 3A,B). What's more, the normalized peak intensities of PAGln were higher in patients with moderate-severe stroke severity and unfavorable outcome at 3 months after stroke (*p* <0.05) (Supplementary Figure S2). The correlation between peak intensities of the top 20 metabolites ranked by VIP values and clinical variables were shown in Figure 3C. PAGln levels showed a significant positive correlation of 0.260 (*p* = 0.001, Spearman correlation analysis) with mRS scores.

### Targeted Metabolomic Analysis Confirmed the Elevation of PAGln Levels in IS Patients

**Figure 4:**
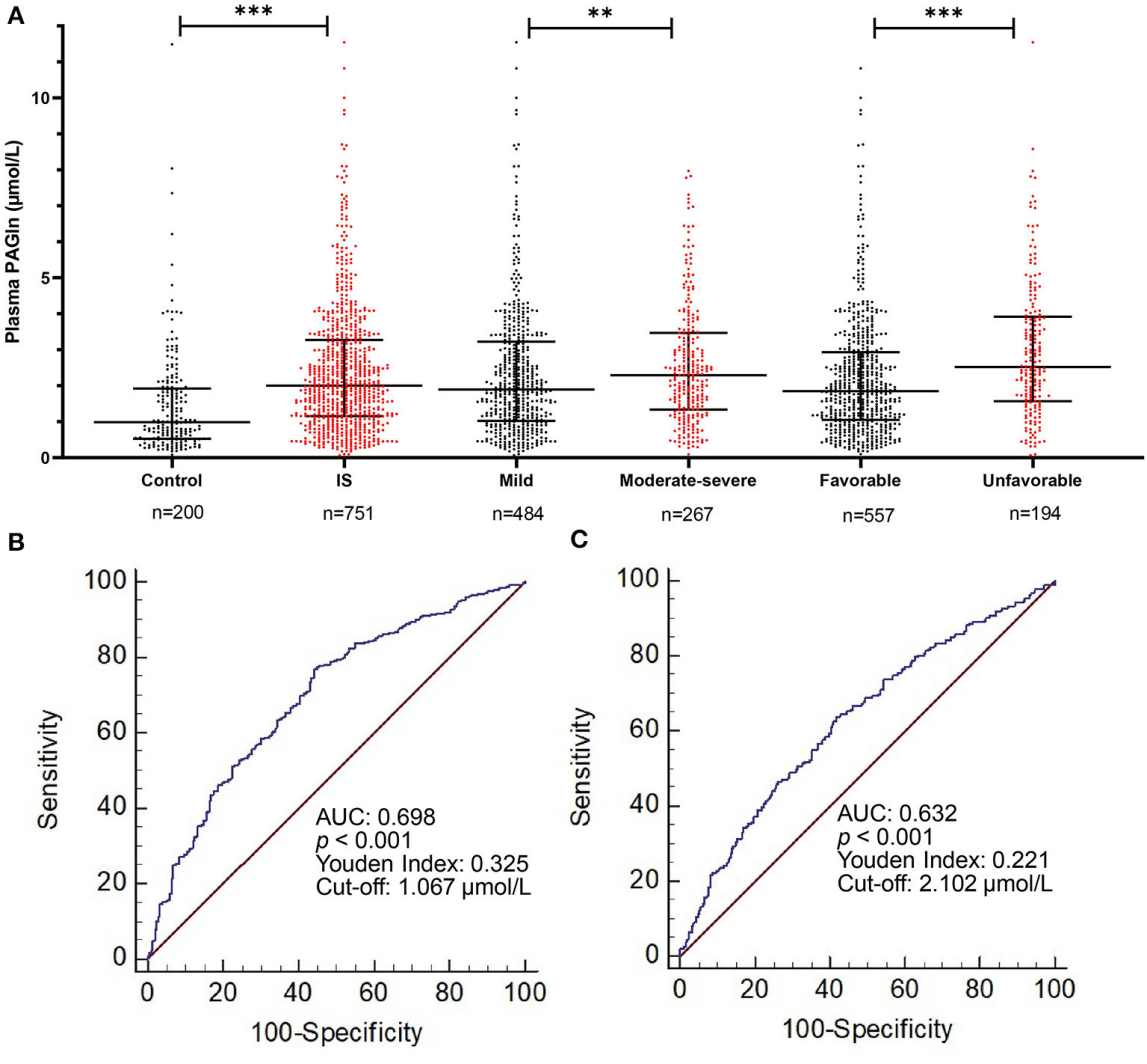
Comparison of plasma PAGln levels between different subject groups. **(A)** PAGln levels in different groups: PAGln levels are higher in IS patients, IS patients with moderate-severe neurological function deficit, and patients with unfavorable short-term outcome (**p < 0.01, ***p < 0.001, Mann–Whitney *U* test). **(B,C)** ROC curves of PAGln levels in predicting IS patients from healthy controls and IS patients with unfavorable outcome from favorable outcome. PAGln, phenylacetylglutamine; IS, ischemic stroke; PAGln, phenylacetylglutamine; ROC, receiver operating characteristic curve; AUC, area under the curve.

**Figure 5:**
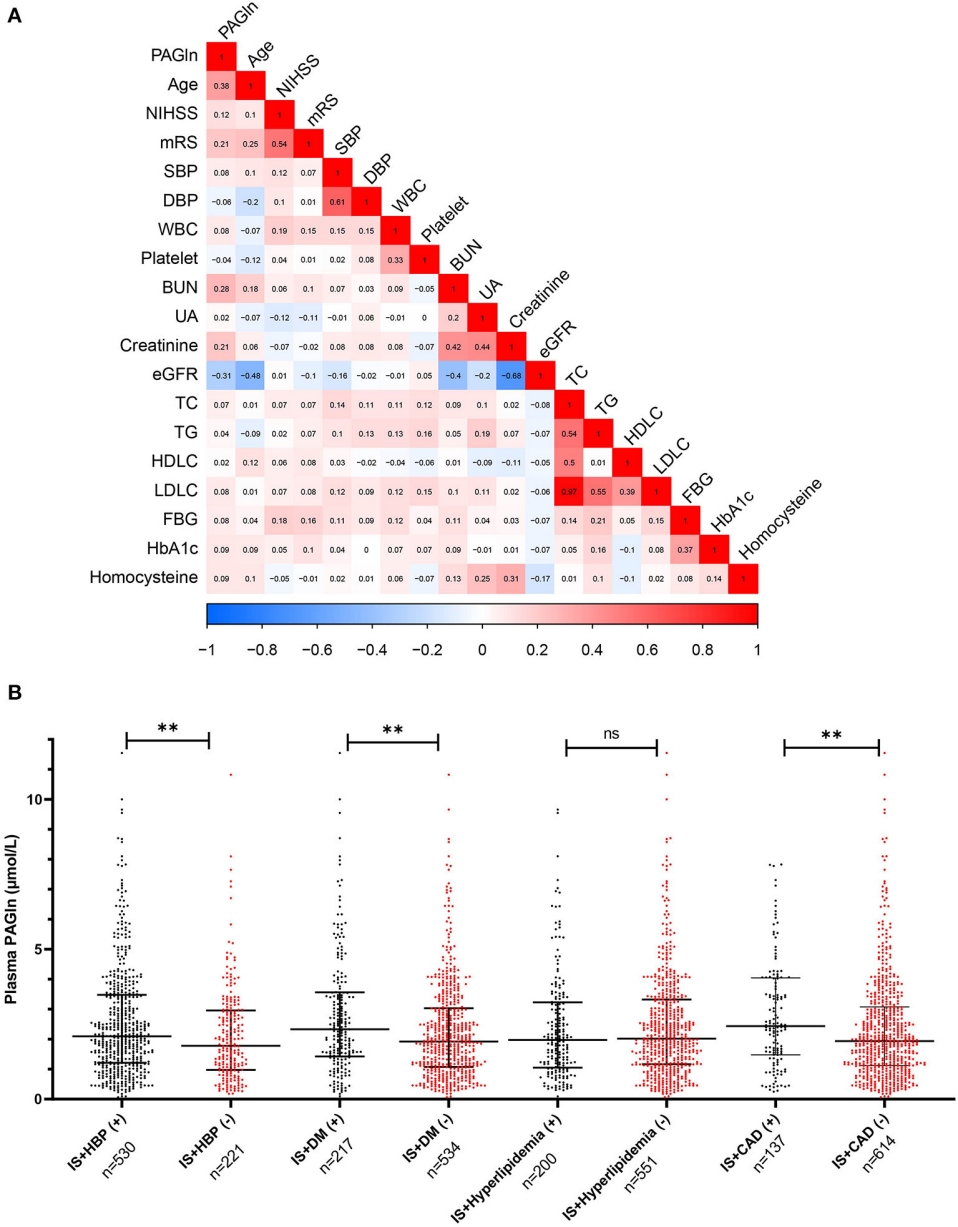
Correlation between PAGln levels and clinical variables in validation stage and PAGln levels in subgroups of vascular risk factors. **(A)** Admission NIHSS scores, mRS scores at 3 months after stroke onset, age, WBC counts, and serum creatinine levels were positively correlated with PAGln levels, eGFR levels showed a negative correlation (Spearman correlation analysis). **(B)** Plasma PAGln levels in patients with or without vascular risk factors. IS patients with HBP had higher PAGln levels than patients without HBP, the same tendencies were also found in patients with DM vs. without DM, and patients with CAD vs. without CAD (**p < 0.01, ***p < 0.001, Mann–Whitney *U* test). IS, ischemic stroke; NIHSS, NIH Stroke Scale; mRS, modified Rankin Scale; WBC: white blood cell; HbA1C, glycosylated hemoglobin A1c; eGFR, estimated glomerular filtration rate; HBP, hypertension; DM, Diabetes mellitus; CAD, coronary artery disease.

Untargeted metabolomic profiling is semiquantitative and requires quantitative analysis. To validate our results of untargeted metabolomics, we analyzed PAGln in an independent cohort of 751 patients with IS and 200 healthy controls. The elevation of PAGln levels in IS patients compared with healthy controls (2.0 [1.2–3.3] μmol/L vs. 1.0 [0.5–1.9] μmol/L, *p* < 0.001; Figure 4A) was confirmed by targeted metabolomics analysis. This significance still existed after adjustments with three models (Table 3). In Model 3, after adjusted for age, sex, hypertension, diabetes mellitus (DM), CAD, hyperlipidemia, smoking, drinking, systolic blood pressure (SBP), white blood cell (WBC) and platelet counts, serum levels of urea nitrogen, uric acid, triglyceride (TG), high-density lipoprotein cholesterol (HDLC), and low-density lipoprotein cholesterol (LDLC), higher levels of PAGln were associated with IS (OR = 3.183, 95% CI 1.671–6.066 for tertile 2 and OR = 9.362, 95% CI 3.797–23.083 for tertile 3, compared with tertile 1, Table 3). The diagnostic value of PAGln in distinguishing patients with IS from healthy controls was evaluated using ROC analysis. The area under the ROC was 0.698 (Figure 4B). The optimal PAGln level cut-off value was 1.067 μmol/L, which yielded a sensitivity of 77.5% and a specificity of 55.0%. We used Spearman correlation analysis to identify the relevance of PAGln levels and clinical parameters (Figure 5A) and found that the admission NIHSS scores, mRS scores at 3 months after stroke onset, age, WBC counts, serum creatinine levels were positively correlated with PAGln levels (Supplementary Table S3 and Supplementary Figure S6), while eGFR levels showed a negative correlation (Supplementary Figure S6).

**Table 3:** Logistic regression analyses of plasma PAGln levels for the occurrence, severity, and function outcome of ischemic stroke.

**PAGln levels** **μmol/L**	**Model 1**			**Model 2**			**Model 3**		
	***p***	**OR**	**95% CI**	***p***	**OR**	**95% CI**	***p***	**OR**	**95% CI**
**IS vs. healthy controls**									
Tertiles									
Tertile 1 (<1.192)	Reference			Reference			Reference		
Tertile 2 (1.192–2.491)	<0.001	3.086	2.104–4.525	<0.001	3.141	2.088–4.724	<0.001	3.183	1.671–6.066
Tertile 3 (> 2.491)	<0.001	6.430	4.030–10.259	<0.001	7.032	4.230–11.689	<0.001	9.362	3.797–23.083
**Moderate–severe vs. mild stroke**									
Tertiles									
Tertile 1 (<1.421)	Reference			Reference			Reference		
Tertile 2 (1.421–2.711)	0.041	1.489	1.017–2.180	0.078	1.415	0.962–2.083	0.686	1.114	0.661–1.878
Tertile 3 (> 2.711)	0.045	1.505	1.009–2.246	0.072	1.452	0.967–2.180	0.374	1.284	0.740–2.228
**Unfavorable vs. favorable outcome**									
Tertiles									
Tertile 1 (<1.421)	Reference			Reference			Reference		
Tertile 2 (1.421–2.711)	0.126	1.428	0.905–2.251	0.251	1.312	0.826–2.084	0.688	1.146	0.590–2.225
Tertile 3 (> 2.711)	0.001	2.190	1.392–3.444	0.003	2.027	1.281–3.209	0.013	2.286	1.188–4.401

***Adjusted model 1:** Adjusted for age and sex (males vs. females)*.

***Adjusted model 2:** Adjusted for age, sex (males vs. females), HBP, DM, hyperlipidaemia, CAD, smoking and drinking status*.

***Adjusted model 3:** IS vs. controls: adjusted for age, sex, HBP, DM, hyperlipidaemia, CAD, smoking, drinking status, SBP, WBC and platelet counts, serum levels of BUN, UA, TG, HDLC, and LDLC*.

*Moderate-severe vs. mild stroke: adjusted for age, sex, HBP, DM, hyperlipidaemia, CAD, smoking, drinking status, SBP, WBC, serum levels of UA, creatinine, TC, HDLC, FBG, HbA1c, and homocysteine*.

*Unfavorable vs. favorable outcome: adjusted for age, sex, HBP, DM, hyperlipidaemia, CAD, smoking, drinking status, admission NIHSS scores, WBC, serum levels of BUN, UA, eGFR, FBG, and HbA1c*.

*PAGln, phenylacetylglutamine; HBP, hypertension; DM, Diabetes mellitus; CAD, coronary artery disease; NIHSS, NIH Stroke Scale; SBP, systolic blood pressure; WBC, white blood cell; BUN: blood urea nitrogen; UA, uric acid; eGFR, estimated glomerular filtration rate; TG, triglyceride; TC, total cholesterol; HDLC, high density lipoprotein cholesterol; LDLC, low density lipoprotein cholesterol; FBG, fasting blood glucose; HbA1c, glycosylated hemoglobin A1c*.

### Relationship Between Plasma PAGln Levels and Stroke Severity of IS Patients

A total of 484 patients (64.4%) had mild stroke and 267 (35.6%) had moderate to severe stroke. The plasma PAGln levels in patients with moderate and severe stroke were higher than in mild stroke patients (2.3 [1.5–3.5] μmol/L vs. 1.9 [1.0–3.2] μmol/L, *p* = 0.007; Figure 4A). This significance still existed after adjusting for age and sex (OR = 1.489, 95% CI 1.017–2.180 for tertile 2 and OR = 1.505, 95% CI 1.009–2.246 for tertile 3, compared with tertile 1, Table 3). After adjusting for other confounding factors in Model 2 and Model 3, plasma PAGln levels were not independently associated with stroke severity (Table 3). When adjusted for age, sex and vascular risk factors (hypertension, DM, hyperlipidemia, CAD, smoking, drinking, one by one), we found that DM was the parameter that resulted in the OR to become not significant. Hypertension and higher baseline WBC counts were significantly associated with moderate-severe stroke.

### Prognostic Value for PAGln in Predicting Unfavorable Outcome of IS Patients

Clinical short-term outcomes of stroke patients were evaluated at 3 months after stroke onset. One hundred and ninety-four patients (25.8%) including 57 (11.8%) patients with mild stroke and 137 (51.3%) patients with moderate-severe stroke eventually developed unfavorable short-term outcomes. The median admission PAGln levels of patients with unfavorable outcomes was higher than in patients with favorable outcomes (2.5 μmol/L vs. 1.9 μmol/L, *p* < 0.001; Figure 4A). Furthermore, in the logistic regression analyses with three models, the highest levels of PAGln were independently associated with unfavorable outcome. In Model 3, after adjusting for age, sex, hypertension, DM, hyperlipidemia, CAD, current smoking and drinking, admission HIHSS scores, WBC counts, serum levels of urea nitrogen, uric acid, eGFR, FBG, and HbA1c, the highest tertile of PAGln showed an OR of 2.286, 95% CI 1.188–4.401, compared with the lowest tertile of PAGln (Table 3). ROC analysis showed that the optimal PAGln level cut-off value for predicting an unfavorable outcome was 2.102 μmol/L, with a sensitivity of 63.7%, specificity of 58.4%, and AUC of 0.632 (Figure 4C).

### Subgroup Analyses of the Association Between PAGln and Vascular Risk Factors

Figure 5B shows the plasma PAGln levels in patients with or without vascular risk factors. The Mann–Whitney *U* tests indicated that IS patients with hypertension had higher PAGln levels than patients without hypertension, the same tendencies were also shown in patients with DM vs. without DM, and patients with CAD vs. without CAD. The logistic regression analyses adjusted by three models in different subgroups were presented in Supplementary Table S4. Higher levels of PAGln were associated with IS with DM in three models (OR = 2.093, 95% CI 1.274–3.437 for tertile 2 and OR = 2.394, 95% CI 1.425–4.023 for tertile 3, compared with tertile 1, Model 3, Supplementary Table S4).

## Discussion

In this study, we implemented a two-stage metabolomic analysis to expand the discovery of metabolites associated with IS. We detected 120 plasma differential metabolites and four metabolic pathways associated with IS. The gut microbiota-dependent metabolite PAGln was significantly higher in patients with IS than in healthy controls in both the discovery and validation stages. Moreover, elevated plasma PAGln levels were shown to be an independent predictor of unfavorable outcomes at 3 months after stroke onset.

With the development of “omics” sciences, many novel biomarkers to bolster the therapeutic targets for IS have been found (15). Metabolomics has emerged as a promising approach to identify potential biomarkers of cerebrovascular disease (2). Many studies have identified differential metabolic biomarkers and pathways associated with stroke risk prediction, early diagnosis, prognosis, and post-stroke depression or cognitive impairment (3, 4, 16). The differential metabolites in our study are shown in Supplementary Table S1; we found that (9Z)-12,13-dihydroxyoctadec-9-enoic acid (12,13-DHOME) reached an AUC of 0.820 to discriminate IS from controls. 12,13-DHOME is a bacterially produced lipid metabolite that serves as a peroxisome proliferator-activated receptor (PPAR) ligand and a potential leukotoxin which can inhibit mitochondrial function (17) and increase oxidative stress in mammals (18). Oxidative stress is one of the most crucial mechanisms induced by ischemia and reperfusion in acute cerebral ischemia phase and might be related to elevated levels of 12,13-DHOME. Taurine, which has antioxidant activity and can combat oxidative stress by regulating the production of reactive oxygen species in mitochondria (19), was shown to be decreased in patients with IS. As seen in Figure 1C, the most markedly affected metabolic pathways in the IS group mainly focused on purine metabolism, TCA cycle, steroid hormone biosynthesis, and pantothenate and CoA biosynthesis. As evidenced by previous studies, purine metabolism is disturbed in patients with IS (20). In our study, levels of inosine and hypoxanthine belonging to purine metabolism were found to be increased in IS patients. Studies have hypothesized that purine metabolism may be related to oxidative stress and that it occurs after ischemic injury (16). Cerebral energy disturbance is an important pathophysiological cascade after IS, and dysregulation of the TCA cycle after stroke is in line with previous studies (21, 22). The pantothenate and CoA biosynthesis pathways play an essential role in energy metabolism and the TCA cycle, which could contribute to ischemic pathophysiology (23). Steroid hormone biosynthesis pathway disturbance was also found in hypoxic-ischemic encephalopathy, which might be due to the response to stress (24).

Several studies have elucidated the role of microbial metabolites in CVD and stroke, such as TMAO and lipopolysaccharide (25, 26). These toxic substances could elicit cardio-cerebrovascular diseases by influencing thrombosis, inflammation, and oxidative stress (27). Among 120 differential metabolites, another gut microbiota-derived metabolite, PAGln, attracted our attention. In comparison with healthy controls, patients with IS showed upregulation of PAGln levels. The normalized peak intensities of PAGln were also higher in patients with severer stroke symptoms and unfavorable outcomes. PAGln, which originates from essential amino acid phenylalanine (28), has been well studied as a uremic toxin in chronic kidney disease (CKD). Recently, one study integrated untargeted and targeted metabolomics with functional metabolomic analysis and identified a novel biomarker associated with CVD risk. They also found that PAGln plays a crucial regulatory role in platelet reactivity and thrombosis via adrenergic receptors in *in vitro* and *in vivo* experiments (7). However, the relationship between PAGln and IS has not been well studied. We then selected PAGln for the subsequent validation study and identified its role in IS. Targeted metabolomics analysis confirmed the elevated levels of PAGln in patients with IS, even after adjusting for potential confounders. Moreover, elevated PAGln levels were observed in patients with more severe IS symptoms. However, this difference was lost after accounting for multiple risk factors. Additionally, our data suggest that higher PAGln levels upon admission may be an independent biomarker of unfavorable short-term outcomes in patients with IS. When just adjusted for age and sex, levels of PAGln were still higher among patients with moderate-severe stroke, comparing to patients with mild stroke. However, after adjusting for clinical confounders, the association was no longer statistically significant, which may be influenced by the presence of DM.

The present study provides new information regarding PAGln levels and IS. Recent untargeted and target metabolomics studies showed that PAGln was associated with an increased risk of CAD and degree of coronary atherosclerotic severity (29, 30). Another targeted metabolomics study demonstrated that high serum PAGln levels are a strong risk factor for future CVD in patients with CKD (31). Circulating PAGln levels were also strongly associated with pulse-wave velocity (an indicator of arterial stiffness) in women (32). Other studies have reported an association between elevated urinary levels of PAGln and obesity (33) and diabetes (34). Accordingly, it is postulated that the association between PAGln and IS could be driven by these risk factors of atherosclerosis. The role of PAGln in regulating platelets and thrombosis may lead to poor outcomes in patients with IS. Notably, our correlation analysis showed that the concentration of PAGln in plasma was positively correlated with age, WBC counts, and serum levels of creatinine, while negatively correlated with eGFR. In line with a previous study, circulating PAGln levels were associated with age and eGFR (calculated using the CKD-EPI formula) (31). Higher plasma PAGln levels among IS patients with diabetes mellitus were also in agreement with a previous study (7). The positive correlation between PAGln and WBC counts indicated that PAGln might be involved in inflammation, thus contributing to the physiological and pathological processes of IS.

There are some limitations to our study. First, the observational nature of the study design cannot prove a causal relationship between PAGln and IS; a functional metabolomics strategy is needed to characterize the underlying mechanisms. Second, the participants in this study belonged to the Chinese Han population, thus, validation of these findings in other populations is required. Third, our study was a single-center study and had a patient selection bias; multi-center validation is necessary in the future. Fourth, PAGln can be influenced by diet and gut microbiota, and information on dietary habits and investigations of the gut microbiome are lacking. Lastly, due to research fund limitations, quantitative analyses of other differential metabolites were not performed; these metabolites will be validated in future studies.

## Conclusions

In summary, our study demonstrated that plasma PAGln levels at admission were elevated in patients with IS and associated with unfavorable short-term outcomes, supporting the hypothesis that PAGln may play a critical role in IS pathogenesis and providing new insight into the interventional target of IS.

## Data Availability Statement

The original contributions presented in the study are included in the article/Supplementary Material, further inquiries can be directed to the corresponding author.

## Ethics Statement

The studies involving human participants were reviewed and approved by Ethics Committee of Xiangya Hospital. The patients/participants provided their written informed consent to participate in this study.

## Author Contributions

JX and BX: conceptualization. FY, YL, MW, and TZ: methodology. FY and XL: software. XF: validation and formal analysis. FY: writing-original draft preparation. JX: writing-review and editing. All authors approved the final version to be published and read and agreed to the published version of the manuscript.

## Funding

This study was supported by the Project Program of National Clinical Research Center for Geriatric Disorders (Xiangya Hospital, Grant No. 2020LNJJ16), the National Natural Science Foundation of China (Grant No. 81671166), the Fundamental Research Funds for the Central Universities of Central South University (Grant No. 2019zzts902), and the Provincial Key Plan for Research and Development of Hunan (Grant No. 2020SK2067).

## Conflict of Interest

The authors declare that the research was conducted in the absence of any commercial or financial relationships that could be construed as a potential conflict of interest.

## Publisher's Note

All claims expressed in this article are solely those of the authors and do not necessarily represent those of their affiliated organizations, or those of the publisher, the editors and the reviewers. Any product that may be evaluated in this article, or claim that may be made by its manufacturer, is not guaranteed or endorsed by the publisher.
