# Supplementary material for: Phenylacetylglutamine, a Novel Biomarker in Acute Ischemic Stroke
Source: Front Cardiovasc Med. 2021 Dec 23;8:798765. doi: 10.3389/fcvm.2021.798765 (PMC8733610; doi:10.3389/fcvm.2021.798765)
Supplement: Supplementary file 1 [file Table_1.DOCX]

**Table S1 Differential metabolites between ischemic stroke patients and healthy controls**

| **Compound** | **HMDB** | **KEGG** | **Formula** | **mz** | **RT (s)** | **Adduct** | **Mode** | ***p*** | **FDR** | **VIP** | **FC** | **AUC** |
| --- | --- | --- | --- | --- | --- | --- | --- | --- | --- | --- | --- | --- |
| 12,13-DHOME | HMDB0004705 | C14829 | C18H34O4 | 313.2386 | 761.9115 | M-H | Negative | <0.001 | <0.001 | 2.953 | 2.971 | 0.820 |
| 9,10-DHOME | HMDB0004704 | C14828 | C18H34O4 | 313.2386 | 761.9115 | M-H | Negative | <0.001 | <0.001 | 2.953 | 2.971 | 0.820 |
| Taurine | HMDB0000251 | C00245 | C2H7NO3S | 124.0070 | 48 | M-H | Negative | <0.001 | <0.001 | 2.867 | 0.651 | 0.821 |
| trans,trans-Farnesyl diphosphate | HMDB0000961 | C00448 | C15H28O7P2 | 381.1190 | 451.954 | M-H | Negative | <0.001 | <0.001 | 2.824 | 5.374 | 0.833 |
| L-Glutamine | HMDB0000641 | C00064 | C5H10N2O3 | 145.0624 | 50.9035 | M-H | Negative | <0.001 | <0.001 | 2.527 | 0.833 | 0.782 |
| 4-(3-Hydroxy-2-naphthyl)-2-oxobut-3-enoic acid | METPA1257 | C16210 | C14H10O4 | 243.0625 | 476.396 | M+H | Positive | <0.001 | <0.001 | 2.488 | 2.993 | 0.813 |
| cis-4-(1'-Hydroxynaphth-2'-yl)-2-oxobut-3-enoate | METPA1025 | C11426 | C14H10O4 | 243.0625 | 476.396 | M+H | Positive | <0.001 | <0.001 | 2.488 | 2.993 | 0.813 |
| Inosine | HMDB0000195 | C00294 | C10H12N4O5 | 267.0735 | 363.325 | M-H | Negative | <0.001 | <0.001 | 2.175 | 1.997 | 0.726 |
| Indole | HMDB0000738 | C00463 | C10H10N2O | 173.0726 | 480.476 | M-H | Negative | <0.001 | <0.001 | 2.169 | 3.537 | 0.803 |
| 2-Amino-3-carboxymuconate semialdehyde | HMDB0001330 | C04409 | C10H12O3 | 203.0696 | 476.406 | M+Na | Positive | <0.001 | <0.001 | 2.154 | 2.192 | 0.765 |
| 2-Hydroxy-3-phenylpropenoate | HMDB0012225 | C02763 | C9H8O3 | 199.0072 | 482.643 | M+Cl | Negative | <0.001 | <0.001 | 2.123 | 0.411 | 0.727 |
| Hypoxanthine | HMDB0000157 | C00262 | C5H4N4O | 137.0460 | 72 | M+H | Positive | <0.001 | <0.001 | 2.103 | 1.579 | 0.686 |
| L-2-Aminoadipate adenylate | HMDB0006941 | C05560 | C16H23N6O10P | 529.0894 | 485.806 | M+K | Positive | <0.001 | <0.001 | 2.068 | 2.373 | 0.713 |
| 3-Dehydrosphinganine | HMDB0001480 | C02934 | C18H37NO2 | 300.2897 | 636.699 | M+H | Positive | <0.001 | <0.001 | 2.040 | 0.724 | 0.725 |
| 5'-Benzoylphosphoadenosine | METPA0821 | C06433 | C17H18N5O8P | 490.0495 | 483.936 | M+K | Positive | <0.001 | <0.001 | 2.037 | 1.956 | 0.706 |
| Dephospho-CoA | HMDB0001373 | C00882 | C21H35N7O13P2S | 688.1699 | 385.0765 | M+H | Positive | <0.001 | <0.001 | 2.022 | 0.582 | 0.771 |
| (Indol-3-yl) acetamide | METPA0317 | C02693 | C10H10N2O | 175.0862 | 482.73 | M+H | Positive | <0.001 | <0.001 | 1.994 | 3.637 | 0.738 |
| Catechol | NA | C00090 | C6H6O2 | 109.0303 | 387.223 | M-H | Negative | <0.001 | <0.001 | 1.987 | 0.575 | 0.710 |
| Hydroquinone | HMDB0002434 | C00530 | C6H6O2 | 109.0303 | 387.223 | M-H | Negative | <0.001 | <0.001 | 1.987 | 0.575 | 0.710 |
| trans-Aconitic acid | HMDB0000958 | C02341 | C6H6O6 | 173.0090 | 84 | M-H | Negative | <0.001 | <0.001 | 1.931 | 0.789 | 0.698 |
| L-Xylulose | HMDB0000751 | C00312 | C5H10O5 | 151.0635 | 373.779 | M+H | Positive | <0.001 | <0.001 | 1.924 | 1.378 | 0.702 |
| Xylose | HMDB0000098 | C00181 | C5H10O5 | 151.0635 | 373.779 | M+H | Positive | <0.001 | <0.001 | 1.924 | 1.378 | 0.702 |
| Phenylacetylglutamine | HMDB0006344 | C04148 | C13H16N2O4 | 265.1183 | 412.1335 | M+H | Positive | <0.001 | <0.001 | 1.923 | 1.968 | 0.697 |
| 1,2-Bis(4-hydroxyphenyl)-2-propanol | METPA1114 | C13629 | C15H16O3 | 267.1090 | 423.385 | M+Na | Positive | <0.001 | <0.001 | 1.912 | 1.484 | 0.706 |
| N-Acetylputrescine | HMDB0002064 | C02714 | C6H14N2O | 131.1175 | 413.0885 | M+H | Positive | <0.001 | <0.001 | 1.877 | 1.475 | 0.704 |
| L-Citrulline | HMDB0000904 | C00327 | C6H13N3O3 | 174.0885 | 53.604 | M-H | Negative | <0.001 | <0.001 | 1.859 | 0.781 | 0.698 |
| 20-COOH-Leukotriene B4 | HMDB0006059 | C05950 | C20H30O6 | 384.2356 | 440.818 | M+NH4 | Positive | <0.001 | <0.001 | 1.847 | 2.334 | 0.696 |
| Androsta-1,4-diene-3,17-dione | HMDB0003422 | C20144 | C19H24O2 | 285.1878 | 550.191 | M+H | Positive | <0.001 | <0.001 | 1.844 | 0.623 | 0.669 |
| Presqualene diphosphate | HMDB0001278 | C03428 | C30H52O7P2 | 625.2841 | 864.761 | M+K | Positive | <0.001 | <0.001 | 1.836 | 0.674 | 0.700 |
| Ornithine | HMDB0000214 | C00077 | C5H12N2O2 | 131.0830 | 53.527 | M-H | Negative | <0.001 | <0.001 | 1.807 | 0.803 | 0.692 |
| Pyruvic acid | HMDB0000243 | C00022 | C3H4O3 | 87.0090 | 72 | M-H | Negative | <0.001 | <0.001 | 1.804 | 0.777 | 0.686 |
| Anisole | HMDB0033895 | C01403 | C7H8O | 107.0511 | 451.379 | M-H | Negative | <0.001 | <0.001 | 1.799 | 2.404 | 0.708 |
| Benzyl alcohol | HMDB0003119 | C03485 | C7H8O | 107.0511 | 451.379 | M-H | Negative | <0.001 | <0.001 | 1.799 | 2.404 | 0.708 |
| Isopentenyladenosine-5'-diphosphate | METPA1687 | C16426 | C15H23N5O10P2 | 518.0831 | 485.021 | M+Na | Positive | <0.001 | <0.001 | 1.789 | 2.420 | 0.701 |
| trans-Zeatin riboside | HMDB0030388 | C16431 | C15H23N5O11P2 | 534.0759 | 487.245 | M+Na | Positive | <0.001 | <0.001 | 1.780 | 2.079 | 0.692 |
| Ajmaline | HMDB0015495 | C06542 | C20H26N2O2 | 349.1832 | 395.131 | M+Na | Positive | <0.001 | <0.001 | 1.779 | 3.626 | 0.713 |
| Phenylacetylglutamine | HMDB0006344 | C04148 | C13H16N2O4 | 263.1041 | 409.0855 | M-H | Negative | <0.001 | <0.001 | 1.770 | 1.849 | 0.700 |
| CMP-N-glycoloylneuraminate | HMDB0012206 | C03691 | C15H25N5O15P2 | 595.1291 | 490.398 | M+NH4 | Positive | <0.001 | <0.001 | 1.755 | 2.115 | 0.690 |
| 11beta,21-Dihydroxy-5beta-pregnane-3,20-dione | HMDB0006757 | C05475 | C21H32O4 | 371.2281 | 401.138 | M+Na | Positive | <0.001 | <0.001 | 1.751 | 10.739 | 0.727 |
| 3alpha,21-Dihydroxy-5beta-pregnane-11,20-dione | HMDB0006755 | C05478 | C21H32O4 | 371.2281 | 401.138 | M+Na | Positive | <0.001 | <0.001 | 1.751 | 10.739 | 0.727 |
| Citraconic acid | HMDB0000634 | C02226 | C5H6O4 | 129.0190 | 78 | M-H | Negative | <0.001 | <0.001 | 1.750 | 0.721 | 0.692 |
| Itaconic acid | HMDB0002092 | C00490 | C5H6O4 | 129.0190 | 78 | M-H | Negative | <0.001 | <0.001 | 1.750 | 0.721 | 0.692 |
| Cortisol | HMDB0000063 | C00735 | C23H32O6 | 422.2602 | 463.211 | M+NH4 | Positive | <0.001 | <0.001 | 1.745 | 2.129 | 0.704 |
| Methyl salicylate | HMDB0029817 | NA | C8H8O3 | 187.0075 | 451.378 | M+Cl | Negative | <0.001 | <0.001 | 1.738 | 2.218 | 0.700 |
| 13(S)-HPOT | METPA0543 | C04785 | C18H30O4 | 328.2481 | 655.089 | M+NH4 | Positive | <0.001 | <0.001 | 1.735 | 0.893 | 0.662 |
| 9(S)-HPOT | METPA1278 | C16321 | C18H30O4 | 328.2481 | 655.089 | M+NH4 | Positive | <0.001 | <0.001 | 1.735 | 0.893 | 0.662 |
| Nicotine | HMDB0014330 | C16150 | C10H14N2 | 163.1326 | 469.232 | M+H | Positive | <0.001 | <0.001 | 1.732 | 0.940 | 0.695 |
| Sphinganine 1-phosphate | HMDB0001383 | C01120 | C18H40NO5P | 380.2569 | 719.209 | M-H | Negative | <0.001 | <0.001 | 1.715 | 0.813 | 0.661 |
| 3,4-Dihydroxybenzoate | HMDB0001856 | C00230 | C7H6O4 | 153.0196 | 438.028 | M-H | Negative | <0.001 | <0.001 | 1.709 | 0.683 | 0.691 |
| Formyl-5-hydroxykynurenamine | HMDB0012948 | C05647 | C10H12N2O3 | 243.0623 | 213.162 | M+Cl | Negative | <0.001 | <0.001 | 1.707 | 0.758 | 0.708 |
| L-Leucine | HMDB0000687 | C00123 | C6H13NO2 | 132.1019 | 184.215 | M+H | Positive | <0.001 | <0.001 | 1.689 | 1.310 | 0.674 |
| trans-o-Hydroxybenzylidenepyruvate | METPA0788 | C06203 | C10H8O4 | 191.0351 | 462.624 | M-H | Negative | <0.001 | 0.001 | 1.668 | 1.979 | 0.714 |
| Salicyluric acid | HMDB0000840 | C07588 | C9H9NO4 | 194.0462 | 440.877 | M-H | Negative | <0.001 | 0.001 | 1.643 | 4.229 | 0.688 |
| Isocitrate | HMDB0000193 | C00311 | C6H8O7 | 191.0199 | 134.558 | M-H | Negative | <0.001 | 0.002 | 1.638 | 0.747 | 0.673 |
| 3-Methyl-2-oxobutanoic acid | HMDB0000019 | C00141 | C5H8O3 | 115.0409 | 374.007 | M-H | Negative | <0.001 | 0.002 | 1.636 | 1.152 | 0.669 |
| 20-Hydroxyleukotriene E4 | HMDB0012639 | C03577 | C23H37NO6S | 490.1970 | 510.279 | M+Cl | Negative | <0.001 | 0.002 | 1.624 | 0.600 | 0.657 |
| 2-Fluorocyclohexadiene-cis,cis-1,2-diol-1-carboxylate | METPA1308 | C16482 | C7H7FO4 | 175.0385 | 463.866 | M+H | Positive | <0.001 | 0.002 | 1.611 | 2.158 | 0.693 |
| 3-Hydroxybutyric acid | HMDB0000357 | C01089 | C4H8O3 | 103.0400 | 96 | M-H | Negative | <0.001 | 0.002 | 1.602 | 1.824 | 0.686 |
| Aflatoxin B2 | HMDB0035208 | C16753 | C17H14O6 | 315.0795 | 375.577 | M+H | Positive | <0.001 | 0.002 | 1.599 | 2.667 | 0.692 |
| Guanine | HMDB0000132 | C00242 | C5H5N5O | 152.0564 | 372.335 | M+H | Positive | <0.001 | 0.002 | 1.597 | 0.921 | 0.757 |
| Adenine | HMDB0000034 | C00147 | C5H5N5 | 136.0615 | 371.478 | M+H | Positive | <0.001 | 0.002 | 1.596 | 0.958 | 0.677 |
| L-Glutamine | HMDB0000641 | C00064 | C5H10N2O3 | 147.0761 | 51.772 | M+H | Positive | <0.001 | 0.002 | 1.590 | 0.895 | 0.669 |
| (9Z)-Hexadecenoic acid | HMDB0003229 | C08362 | C16H30O2 | 253.2175 | 1091.07 | M-H | Negative | <0.001 | 0.003 | 1.561 | 1.538 | 0.640 |
| P1,P4-Bis(5'-guanosyl) tetraphosphate | HMDB0001340 | C01261 | C20H28N10O21P4 | 869.0468 | 496.071 | M+H | Positive | <0.001 | 0.003 | 1.537 | 0.703 | 0.680 |
| 5-Hydroxyisourate | HMDB0030097 | C11821 | C5H4N4O4 | 218.9970 | 417.795 | M+Cl | Negative | <0.001 | 0.004 | 1.510 | 0.383 | 0.696 |
| ITP | HMDB0000189 | C00081 | C10H15N4O14P3 | 508.9949 | 472.147 | M+H | Positive | <0.001 | 0.004 | 1.507 | 2.018 | 0.653 |
| N-(5-Phospho-D-ribosyl)anthranilate | METPA0483 | C04302 | C12H16NO9P | 350.0668 | 515.287 | M+H | Positive | <0.001 | 0.004 | 1.504 | 0.858 | 0.669 |
| ADP-5-ethyl-4-methylthiazole-2-carboxylate | NA | C20784 | C17H22N6O12P2S | 579.0359 | 504.57 | M+H-H2O | Positive | <0.001 | 0.004 | 1.495 | 1.675 | 0.651 |
| Salicin 6-phosphate | METPA0784 | C06188 | C13H19O10P | 367.0812 | 492.972 | M+H | Positive | <0.001 | 0.005 | 1.493 | 2.868 | 0.607 |
| 2-Furoic acid | HMDB0000617 | C01546 | C5H4O3 | 111.0090 | 72 | M-H | Negative | <0.001 | 0.005 | 1.492 | 0.877 | 0.657 |
| 4-Methylumbelliferyl acetate | HMDB0032989 | C03837 | C12H10O4 | 217.0510 | 490.745 | M-H | Negative | <0.001 | 0.005 | 1.479 | 2.261 | 0.684 |
| 5-Sulfosalicylate | HMDB0011725 | C16199 | C7H6O6S | 216.9808 | 383.1945 | M-H | Negative | <0.001 | 0.005 | 1.478 | 1.413 | 0.663 |
| L-Malic acid | HMDB0000156 | C00149 | C4H6O5 | 133.0140 | 72 | M-H | Negative | <0.001 | 0.005 | 1.478 | 0.826 | 0.669 |
| Guanosine | HMDB0000133 | C00387 | C10H17N5O17P4 | 603.9733 | 499.2865 | M+H | Positive | <0.001 | 0.005 | 1.470 | 2.106 | 0.685 |
| DL-2-Aminooctanoic acid | HMDB0000991 | NA | C8H17NO2 | 160.1330 | 402 | M+H | Positive | 0.001 | 0.007 | 1.440 | 0.763 | 0.658 |
| (8S)-3',8-Cyclo-7,8-dihydroguanosine 5'-triphosphate | NA | C21310 | C10H16N5O14P3 | 524.0045 | 473.547 | M+H | Positive | 0.001 | 0.007 | 1.435 | 2.016 | 0.650 |
| GTP | HMDB0001273 | C00044 | C10H16N5O14P3 | 524.0045 | 473.547 | M+H | Positive | 0.001 | 0.007 | 1.435 | 2.016 | 0.650 |
| Glutaryl-CoA | HMDB0001339 | C00527 | C26H42N7O19P3S | 882.1536 | 495.3955 | M+H | Positive | 0.001 | 0.007 | 1.428 | 0.693 | 0.663 |
| Citric acid | HMDB0000094 | C00158 | C6H8O7 | 191.0200 | 72 | M-H | Negative | 0.001 | 0.008 | 1.420 | 0.892 | 0.643 |
| Suberic acid | HMDB0000893 | C08278 | C8H14O4 | 173.0824 | 433.718 | M-H | Negative | 0.001 | 0.008 | 1.406 | 1.025 | 0.672 |
| S-(4-Methylthiobutylthiohydroximoyl)-L-cysteine | METPA1743 | C17242 | C8H16N2O3S2 | 275.0487 | 516.185 | M+Na | Positive | 0.002 | 0.008 | 1.403 | 0.805 | 0.644 |
| Corticosterone | HMDB0001547 | C02140 | C21H30O4 | 381.1740 | 580.003 | M+Cl | Negative | 0.002 | 0.009 | 1.399 | 0.771 | 0.652 |
| Phenyl acetate | HMDB0040733 | C15583 | C8H8O2 | 135.0456 | 443.912 | M-H | Negative | 0.002 | 0.009 | 1.397 | 0.808 | 0.647 |
| Pantothenate | HMDB0000210 | C00864 | C9H17NO5 | 218.1036 | 370.7935 | M-H | Negative | 0.002 | 0.009 | 1.395 | 0.804 | 0.630 |
| Feruloyl-CoA | METPA0038 | C00406 | C31H44N7O19P3S | 966.1639 | 496.151 | M+Na | Positive | 0.002 | 0.009 | 1.392 | 0.760 | 0.663 |
| 5alpha-Dihydrodeoxycorticosterone | HMDB0060407 | C18040 | C21H32O3 | 367.1966 | 559.084 | M+Cl | Negative | 0.002 | 0.009 | 1.390 | 1.555 | 0.652 |
| Succinic acid | HMDB0000254 | C00042 | C4H6O4 | 117.0190 | 84 | M-H | Negative | 0.002 | 0.009 | 1.386 | 0.784 | 0.661 |
| 4-Hydroxybenzoate | HMDB0000500 | C00156 | C7H6O3 | 137.0250 | 484.536 | M-H | Negative | 0.002 | 0.009 | 1.381 | 2.740 | 0.643 |
| Salicylate | HMDB0000840 | C07588 | C7H6O3 | 137.0250 | 484.536 | M-H | Negative | 0.002 | 0.009 | 1.381 | 2.740 | 0.643 |
| Linoleate | HMDB0000673 | C01595 | C18H32O2 | 279.2333 | 1114.46 | M-H | Negative | 0.002 | 0.009 | 1.381 | 1.393 | 0.620 |
| Dihydrosterigmatocystin | HMDB0030590 | NA | C18H14O6 | 349.0686 | 412.457 | M+Na | Positive | 0.002 | 0.009 | 1.378 | 1.369 | 0.648 |
| L-Carnitine | HMDB0000062 | C00318 | C7H15NO3 | 162.1120 | 54 | M+H | Positive | 0.002 | 0.011 | 1.355 | 1.149 | 0.641 |
| Allotetrahydrodeoxycorticosterone | HMDB0000879 | C13713 | C21H34O3 | 373.2198 | 554.574 | M+K | Positive | 0.003 | 0.015 | 1.310 | 0.950 | 0.630 |
| 1,7-Dimethylxanthine | HMDB0001860 | C13747 | C7H8N4O2 | 181.0716 | 386.187 | M+H | Positive | 0.003 | 0.016 | 1.302 | 0.283 | 0.571 |
| Theophylline | HMDB0001889 | C07130 | C7H8N4O2 | 181.0716 | 386.187 | M+H | Positive | 0.003 | 0.016 | 1.302 | 0.283 | 0.571 |
| Pantetheine | HMDB0003426 | C00831 | C11H22N2O4S | 277.1229 | 388.3895 | M-H | Negative | 0.003 | 0.017 | 1.296 | 1.113 | 0.656 |
| Anthraniloyl-CoA | NA | C02247 | C28H41N8O17P3S | 887.1479 | 495.0895 | M+H | Positive | 0.004 | 0.018 | 1.284 | 0.754 | 0.644 |
| Paraoxon | HMDB0013035 | C06606 | C10H14NO6P | 293.0814 | 566.876 | M+NH4 | Positive | 0.004 | 0.021 | 1.259 | 0.834 | 0.640 |
| Caffeine | HMDB0001847 | C07481 | C8H10N4O2 | 195.0871 | 403.007 | M+H | Positive | 0.005 | 0.022 | 1.253 | 0.344 | 0.557 |
| Arachidonate | HMDB0060102 | C00219 | C20H32O2 | 303.2332 | 1105.11 | M-H | Negative | 0.005 | 0.023 | 1.245 | 1.225 | 0.598 |
| cis-1,2-Dihydro-3-ethylcatechol | METPA0864 | C06727 | C8H12O2 | 158.1171 | 390.093 | M+NH4 | Positive | 0.005 | 0.023 | 1.245 | 0.808 | 0.631 |
| Phosphonoacetate | HMDB0004110 | C05682 | C2H5O5P | 140.9992 | 609.996 | M+H | Positive | 0.005 | 0.023 | 1.242 | 1.143 | 0.648 |
| 20alpha-Hydroxy-4-pregnen-3-one | HMDB0003069 | C04042 | C21H32O2 | 317.2518 | 509.821 | M+H | Positive | 0.006 | 0.025 | 1.231 | 0.608 | 0.623 |
| Pregnenolone | HMDB0000253 | C01953 | C21H32O2 | 317.2518 | 509.821 | M+H | Positive | 0.006 | 0.025 | 1.231 | 0.608 | 0.623 |
| Dihydroneopterin phosphate | HMDB0006824 | C05925 | C9H14N5O7P | 336.0632 | 479.255 | M+H | Positive | 0.006 | 0.026 | 1.223 | 1.483 | 0.628 |
| Ethylbenzene | HMDB0059905 | C07111 | C8H10 | 107.0858 | 544.0885 | M+H | Positive | 0.006 | 0.028 | 1.213 | 1.061 | 0.698 |
| L-Threonine | HMDB0000167 | C00188 | C4H9NO3 | 118.0516 | 50.9085 | M-H | Negative | 0.007 | 0.033 | 1.188 | 0.874 | 0.638 |
| Deoxyadenosine | HMDB0000101 | C00559 | C10H13N5O3 | 252.1089 | 371.792 | M+H | Positive | 0.008 | 0.034 | 1.180 | 0.966 | 0.626 |
| Aniline | HMDB0003012 | C00292 | C6H7N | 128.0359 | 363.512 | M+Cl | Negative | 0.008 | 0.034 | 1.179 | 1.161 | 0.636 |
| Theophylline | HMDB0001889 | C07130 | C7H8N4O2 | 179.0575 | 379.954 | M-H | Negative | 0.008 | 0.036 | 1.171 | 0.400 | 0.545 |
| 6-Hydroxyhexanoic acid | HMDB0012843 | C06103 | C6H12O3 | 133.0792 | 441.5085 | M+H | Positive | 0.009 | 0.037 | 1.166 | 1.230 | 0.630 |
| 1-(5'-Phosphoribosyl)-5-formamido-4-imidazolecarboxamide | HMDB0001439 | C04734 | C10H15N4O9P | 384.0987 | 489.1525 | M+NH4 | Positive | 0.009 | 0.037 | 1.165 | 3.768 | 0.590 |
| 4-Coumarate | HMDB0002035 | C00811 | C9H8O3 | 163.0403 | 457.837 | M-H | Negative | 0.009 | 0.038 | 1.156 | 0.867 | 0.667 |
| trans-2-Hydroxycinnamate | HMDB0002641 | C01772 | C9H8O3 | 163.0403 | 457.837 | M-H | Negative | 0.009 | 0.038 | 1.156 | 0.867 | 0.667 |
| Stearidonic acid | HMDB0006547 | C16300 | C18H28O2 | 277.2161 | 998.08 | M+H | Positive | 0.010 | 0.040 | 1.149 | 0.823 | 0.615 |
| 17alpha,20alpha-Dihydroxypregn-4-en-3-one | HMDB0011653 | C04518 | C21H32O3 | 333.2464 | 469.385 | M+H | Positive | 0.012 | 0.047 | 1.121 | 0.677 | 0.617 |
| 17alpha-Hydroxypregnenolone | HMDB0000363 | C05138 | C21H32O3 | 333.2464 | 469.385 | M+H | Positive | 0.012 | 0.047 | 1.121 | 0.677 | 0.617 |
| 5alpha-Androstane-3,17-dione | HMDB0000899 | C00674 | C19H28O2 | 289.2201 | 469.455 | M+H | Positive | 0.012 | 0.047 | 1.118 | 0.721 | 0.626 |
| Dehydroepiandrosterone sulfate | HMDB0001032 | C04555 | C19H28O2 | 289.2201 | 469.455 | M+H | Positive | 0.012 | 0.047 | 1.118 | 0.721 | 0.626 |
| Catechol | NA | C00090 | C6H6O2 | 128.0704 | 378.5845 | M+NH4 | Positive | 0.012 | 0.048 | 1.116 | 1.243 | 0.610 |

Abbreviations: HDMB: Human Metabolome Database; KEGG: Kyoto Encyclopedia of Genes and Genomes; RT: retention time; FDR: false discovery rate; FC: fold change: IS/healthy; VIP: variable influence on projection; AUC: area under the ROC curves.

**Table S2 Results of KEGG pathway analysis**

| Pathway | Total | Hits | *p* | FDR | Impact |
| --- | --- | --- | --- | --- | --- |
| Purine metabolism | 65 | 11 | 0.005 | 0.340 | 0.127 |
| Citrate cycle (TCA cycle) | 20 | 5 | 0.012 | 0.340 | 0.259 |
| Steroid hormone biosynthesis | 85 | 12 | 0.016 | 0.340 | 0.144 |
| Pantothenate and CoA biosynthesis | 19 | 4 | 0.043 | 0.722 | 0.250 |

Abbreviations: KEGG: Kyoto Encyclopedia of Genes and Genomes; FDR: false discovery rate.

**Table S3 Correlation between the PAGln levels and clinical** **parameters in IS patients in validation stage.**

| **Variables** | **Plasma PAGln concentrations, µmol/L** | |
| --- | --- | --- |
|  | r | *p* |
| Age, years | 0.38 | **<0.001** |
| NIHSS (on admission) | 0.12 | **0.032** |
| mRS (3 months after stroke) | 0.22 | **<0.001** |
| SBP (mmHg) | 0.08 | 0.148 |
| DBP (mmHg) | -0.06 | 0.401 |
| WBC, ×10^9^/L | 0.08 | **0.018** |
| Platelet, ×10^9^/L | -0.04 | 0.624 |
| BUN (mmol/L) | 0.28 | 0.817 |
| UA (µmol/L) | 0.02 | 0.129 |
| Creatinine (µmol/L) | 0.21 | **<0.001** |
| eGFR, ml/min per1.73 m^2^ | -0.31 | **<0.001** |
| TC (mmol/L) | 0.07 | 0.287 |
| TG (mmol/L) | 0.04 | 0.576 |
| HDLC (mmol/L) | -0.02 | 0.545 |
| LDLC (mmol/L) | 0.08 | 0.057 |
| FBG (mmol/L) | 0.08 | 0.186 |
| HbA1c (%) | 0.09 | 0.061 |
| Homocysteine (µmol/L) | 0.09 | 0.506 |

The correlation between the clinical parameters and PAGln levels was evaluated by the Spearman correlation test.

Abbreviations: PAGln: phenylacetylglutamine; IS: ischemic stroke; NIHSS: National Institutes of Health Stroke Scale; mRS: modified Rankin Scale; SBP: systolic blood pressure, DBP: diastolic blood pressure; WBC: white blood cell; BUN: blood urea nitrogen; UA: uric acid; eGFR: estimated glomerular filtration rate; TC: total cholesterol; TG: triglyceride; HDLC: high density lipoprotein cholesterol; LDLC: low density lipoprotein cholesterol; FBG: fasting blood glucose; HbA1c: glycosylated hemoglobin A1c.

**Table S4 Logistic regression analyses of plasma PAGln levels in subgroups**

| **PAGln levels**  **µmol/L** | **Model 1** | | |  | **Model 2** | | |  | **Model 3** | | |
| --- | --- | --- | --- | --- | --- | --- | --- | --- | --- | --- | --- |
|  | ***p*** | **OR** | **95% CI** |  | ***p*** | **OR** | **95% CI** |  | ***p*** | **OR** | **95% CI** |
| **IS with HBP vs. without HBP** |  |  |  |  |  |  |  |  |  |  |  |
| Tertiles |  |  |  |  |  |  |  |  |  |  |  |
| Tertile 1 (< 1.421) | Reference |  |  |  | Reference |  |  |  | Reference |  |  |
| Tertile 2 (1.421-2.711) | 0.383 | 1.197 | 0.799-1.792 |  | 0.431 | 1.181 | 0.781-1.787 |  | 0.561 | 0.861 | 0.519-1.427 |
| Tertile 3 (> 2.711) | 0.524 | 0.871 | 0.569-1.333 |  | 0.380 | 0.821 | 0.528-1.276 |  | 0.095 | 0.626 | 0.361-1.085 |
| **IS with DM vs. without DM** |  |  |  |  |  |  |  |  |  |  |  |
| Tertiles |  |  |  |  |  |  |  |  |  |  |  |
| Tertile 1 (< 1.421) | Reference |  |  |  | Reference |  |  |  | Reference |  |  |
| Tertile 2 (1.421-2.711) | 0.026 | 1.600 | 1.057-2.422 |  | 0.028 | 1.600 | 1.051-2.436 |  | 0.004 | 2.093 | 1.274-3.437 |
| Tertile 3 (> 2.711) | 0.001 | 2.062 | 1.343-3.167 |  | 0.001 | 2.109 | 1.365-3.258 |  | 0.001 | 2.394 | 1.425-4.023 |
| **IS with CAD vs. without CAD** |  |  |  |  |  |  |  |  |  |  |  |
| Tertiles |  |  |  |  |  |  |  |  |  |  |  |
| Tertile 1 (< 1.421) | Reference |  |  |  | Reference |  |  |  | Reference |  |  |
| Tertile 2 (1.421-2.711) | 0.612 | 1.140 | 0.687-1.890 |  | 0.736 | 1.091 | 0.656-1.816 |  | 0.672 | 1.118 | 0.667-1.873 |
| Tertile 3 (> 2.711) | 0.581 | 1.154 | 0.694-1.917 |  | 0.702 | 1.105 | 0.663-1.842 |  | 0.910 | 1.031 | 0.610-1.741 |

Adjusted model 1: adjusted for age and sex (males vs. females).

IS with HBP vs. without HBP: adjusted model 2: adjusted for age, sex (males vs. females), DM, hyperlipidaemia, CAD, smoking and drinking status; adjusted model 3: adjusted for age, sex, DM, hyperlipidaemia, CAD, smoking, drinking status, WBC, serum levels of BUN, eGFR, UA, FBG, and homocysteine.

IS with DM vs. without DM: adjusted model 2: adjusted for age, sex (males vs. females), HBP, hyperlipidaemia, CAD, smoking and drinking status; adjusted model 3: adjusted for age, sex, HBP, hyperlipidaemia, CAD, smoking, drinking status, SBP, WBC and platelet counts, serum levels of UA, TC, HDLC, and homocysteine.

IS with CAD vs. without CAD: adjusted model 2: adjusted for age, sex (males vs. females), HBP, DM, hyperlipidaemia, smoking and drinking status; adjusted model 3: adjusted for age, sex, HBP, DM, hyperlipidaemia, smoking, drinking status, WBC and serum levels of eGFR.

Abbreviations: PAGln: phenylacetylglutamine; IS: ischemic stroke; HBP: hypertension; DM: Diabetes mellitus; CAD: coronary artery disease; SBP: systolic blood pressure; WBC: white blood cell; BUN: blood urea nitrogen; UA: uric acid; eGFR: estimated glomerular filtration rate; TC: total cholesterol; TG: triglyceride; HDLC: high density lipoprotein cholesterol; FBG: fasting blood glucose.
