# Supplementary material for: Phenylacetylglutamine, a Novel Biomarker in Acute Ischemic Stroke
Source: Front Cardiovasc Med. 2021 Dec 23;8:798765. doi: 10.3389/fcvm.2021.798765 (PMC8733610; doi:10.3389/fcvm.2021.798765)
Supplement: Supplementary file 2 [file Data_Sheet_1.PDF]

Figure S1 Typical liquid chromatography–mass spectrometry (LC–MS) total ion chromatogram (TIC) in positive (POS) and negative (NEG) ion mode of the quality control (QC) plasma samples.

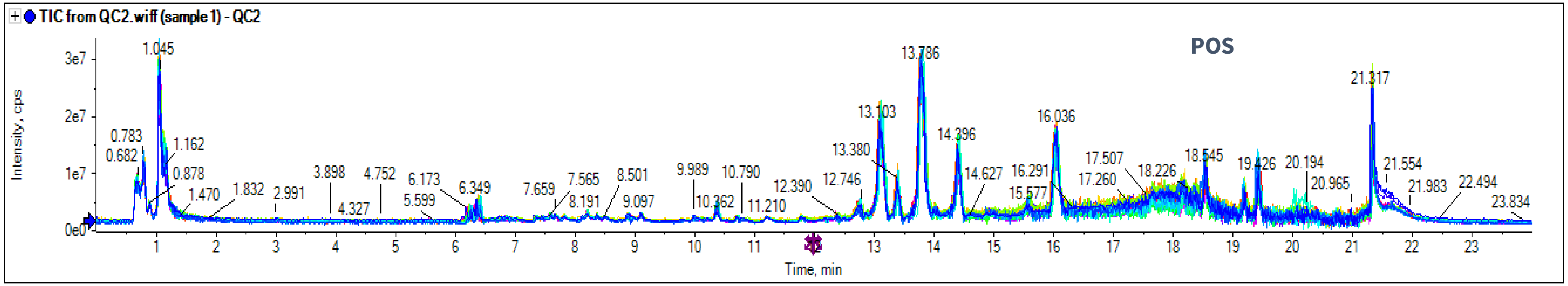

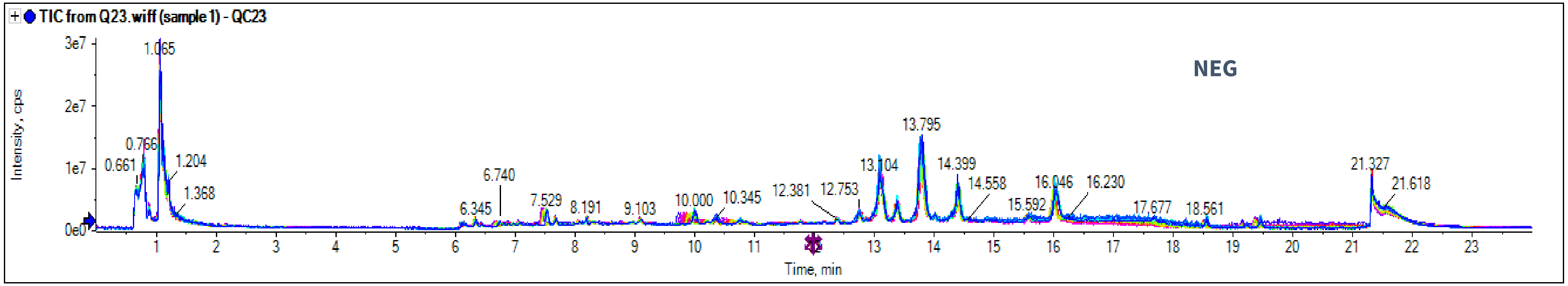

Figure S2 The peak intensity of plasma phenylacetylglutamine (PAGln) and the severity and short-term outcome of patients with ischemic stroke in discovery stage.

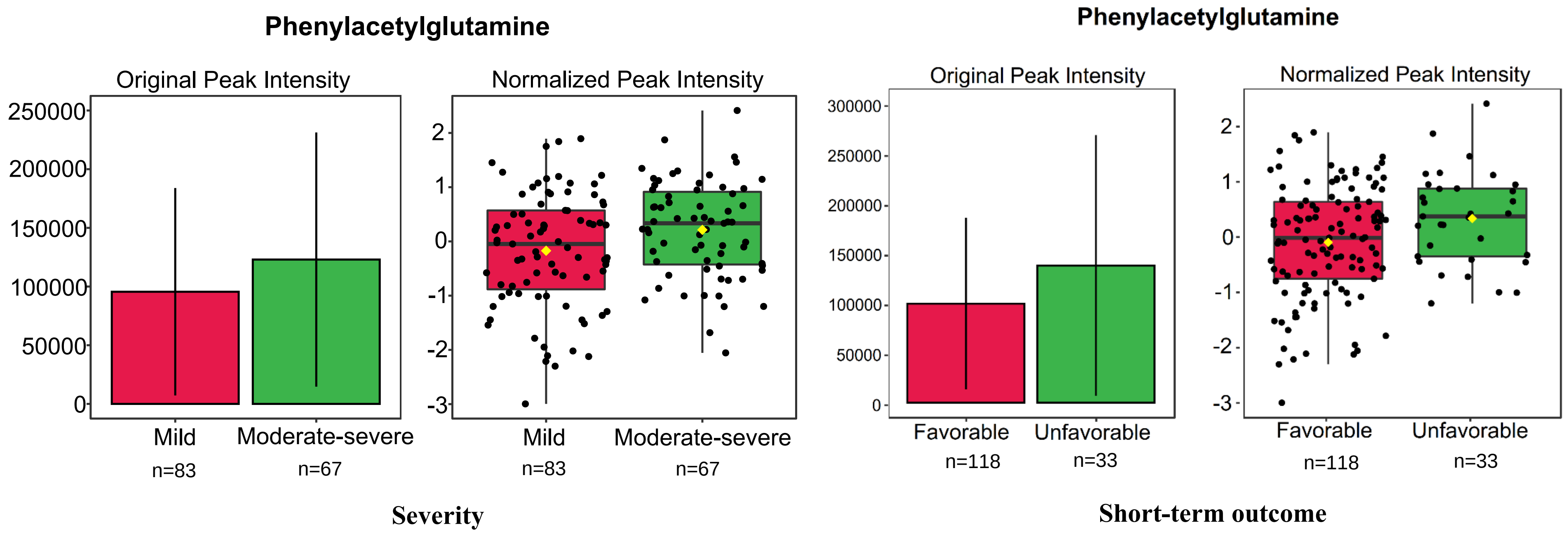

The severity of stroke was assessed by the National Institutes of Health Stroke Scale (NIHSS) score: mild (NIHSS 0-5), moderate to severe (NIHSS >5).
The short-term outcome of stroke patients was assessed by the modified Rankin Scale (mRS) score at three months after stroke onset: favorable (mRS 0-2), unfavorable (mRS 3-6).

Figure S3 Plots of the most important metabolites (top 5, 10, 15, 25) from the Monte-Carlo cross validation analysis

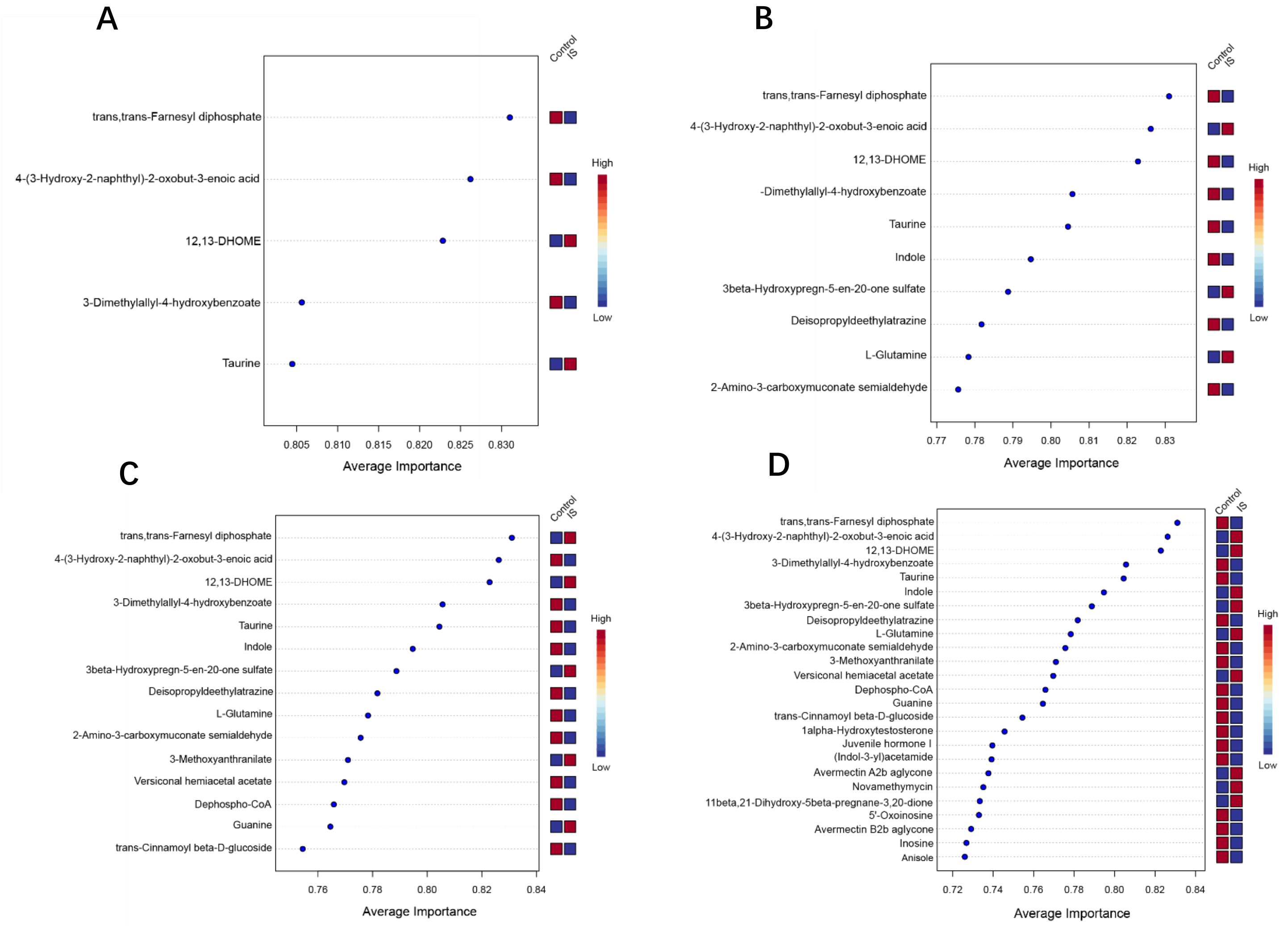

The most important metabolites of Model 1 (A, 5 features), Model 2 (B, 10 features), Model 3 (C, 15 features), and Model 4 (D, 25 features) of the multivariable ROC curves. The X-axis represents the name of metabolites, and the Y-axis represents the mean importance.

Figure S4 The spectrum form of PAGln (m/z of 265.1184) from the discovery dataset.

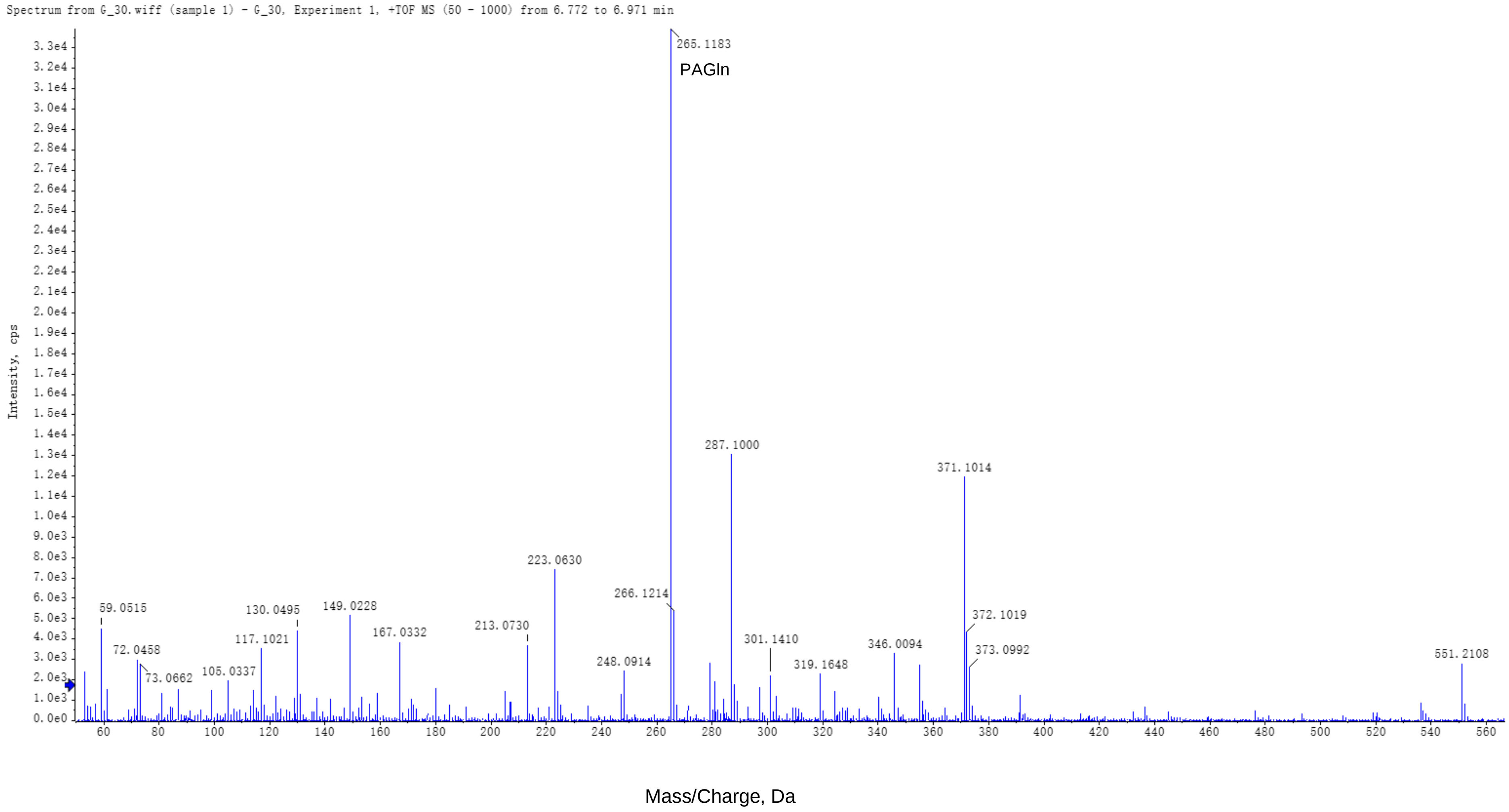

Figure S5 Chromatograms generated by multiple reaction monitoring transitions (MRM) for PAGln (m/z 265.2→130.1) and the isotope internal standard (D5-PAGln) (m/z 270.1→130.1) in positive-ion mode (A) and the linear calibration curves for LC/MS analysis of PAGln (r^2>0.999) with concentrations ranging from 1 to 500ng/ml..

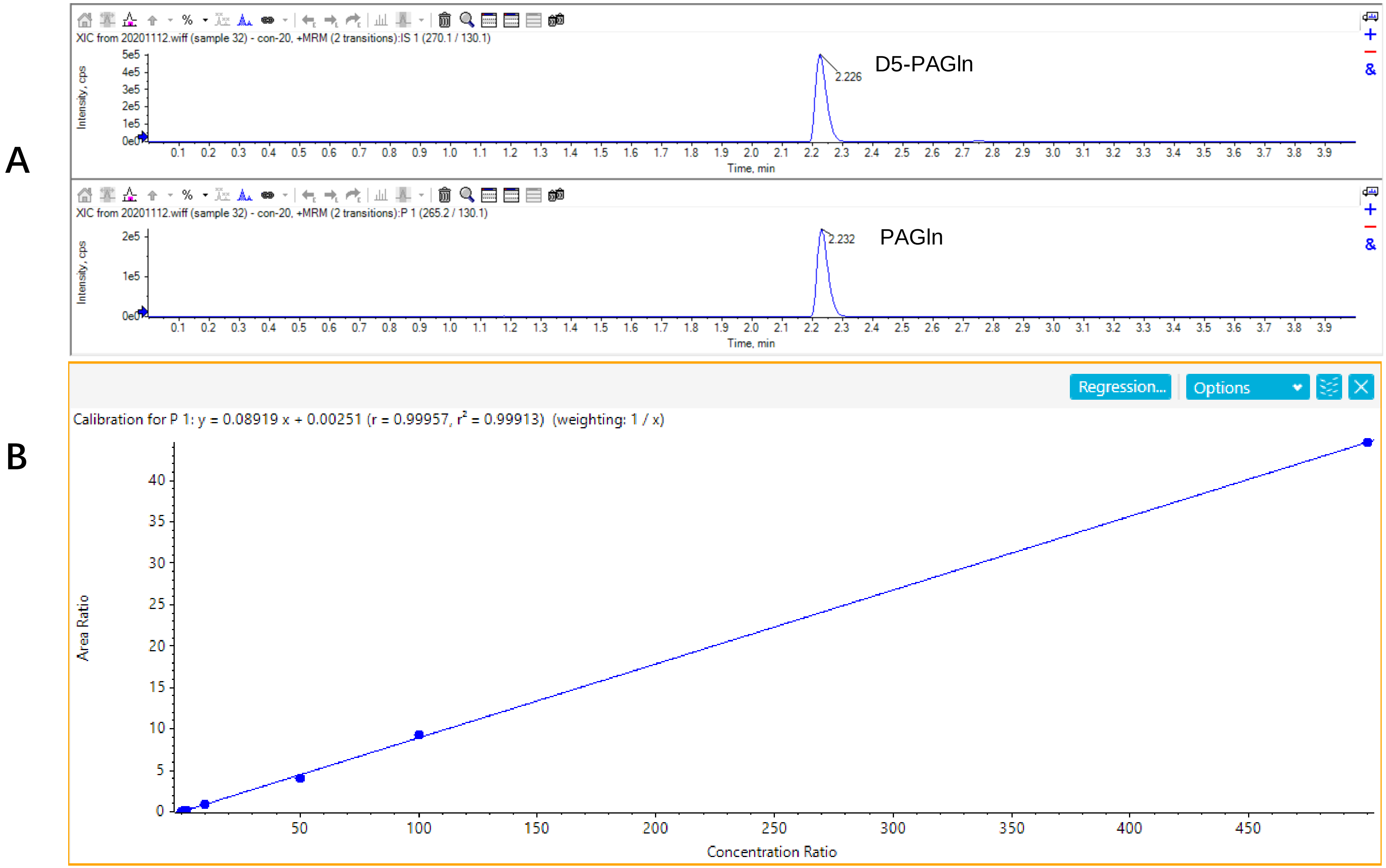

Figure S6 Correlation between PAGln levels and clinical parameters of IS patients in validation stage (Spearman correlation analysis).

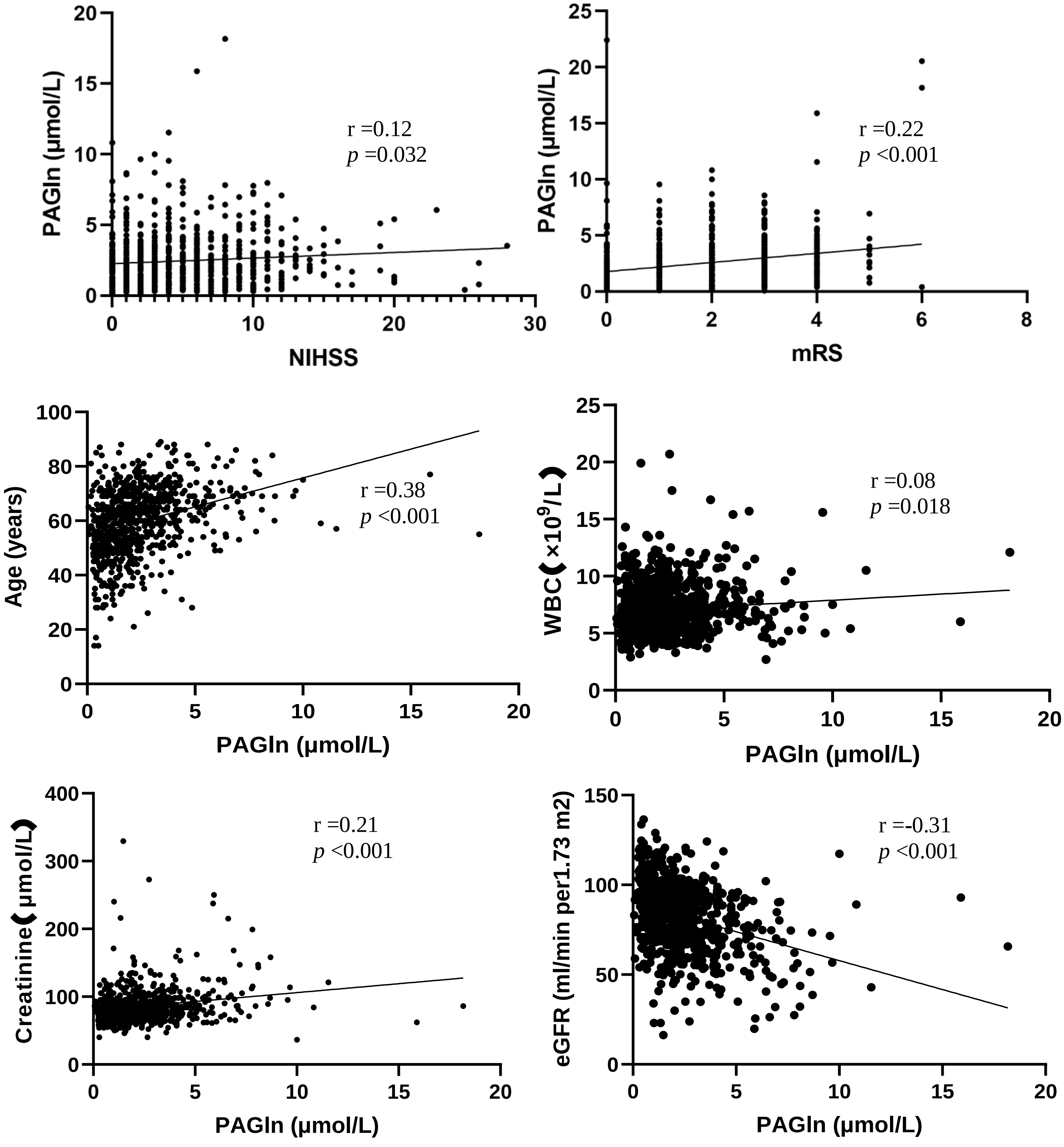

NIHSS: National Institutes of Health Stroke Scale, assessed on admission; mRS: modified Rankin Scale; assessed at three months after stroke onset. PAGln: phenylacetylglutamine; WBC: white blood cell; HbA1C: glycosylated hemoglobin A1c; eGFR: estimated glomerular filtration rate.
